# Comparative genome analyses reveal sequence features reflecting distinct modes of host-adaptation between dicot and monocot powdery mildew

**DOI:** 10.1186/s12864-018-5069-z

**Published:** 2018-09-25

**Authors:** Ying Wu, Xianfeng Ma, Zhiyong Pan, Shiv D. Kale, Yi Song, Harlan King, Qiong Zhang, Christian Presley, Xiuxin Deng, Cheng-I Wei, Shunyuan Xiao

**Affiliations:** 1 Institute for Bioscience and Biotechnology Research, University of Maryland, College Park, MD 20850 USA; 2 Hunan Provincial Key Laboratory for Germplasm Innovation and Utilization of Crop, Hunan Agricultural University, Changsha, 410128 China; 3 Key Laboratory of Horticultural Plant Biology (Ministry of Education), Key Laboratory of Horticultural Crop Biology and Genetic Improvement (Central Region, Ministry of Agriculture), College of Horticulture and Forestry Sciences, Huazhong Agricultural University, Wuhan, 430070 China; 4 Biocomplexity Institute, Virginia Tech, Blacksburg, VA 24061 USA; 5 College of Food Science and Nutritional Engineering, China Agricultural University, Beijing, 100083 China; 6 College of Agriculture & Natural Resources, University of Maryland, College Park, MD 20742 USA; 7 Department of Plant Science and Landscape Architecture, University of Maryland, College Park, MD 20742 USA

**Keywords:** Powdery mildew, Genome comparison, Host adaptation, Effectors, Gene loss, Gene conservation, Host-pathogen arms race

## Abstract

**Background:**

Powdery mildew (PM) is one of the most important and widespread plant diseases caused by biotrophic fungi. Notably, while monocot (grass) PM fungi exhibit high-level of host-specialization, many dicot PM fungi display a broad host range. To understand such distinct modes of host-adaptation, we sequenced the genomes of four dicot PM biotypes belonging to *Golovinomyces cichoracearum* or *Oidium neolycopersici*.

**Results:**

We compared genomes of the four dicot PM together with those of *Blumeria graminis* f.sp. *hordei* (both DH14 and RACE1 isolates)*, B. graminis* f.sp. *tritici*, and *Erysiphe necator* infectious on barley, wheat and grapevine, respectively. We found that despite having a similar gene number (6620–6961), the PM genomes vary from 120 to 222 Mb in size. This high-level of genome size variation is indicative of highly differential transposon activities in the PM genomes. While the total number of genes in any given PM genome is only about half of that in the genomes of closely related ascomycete fungi, most (~ 93%) of the ascomycete core genes (ACGs) can be found in the PM genomes. Yet, 186 ACGs were found absent in at least two of the eight PM genomes, of which 35 are missing in some dicot PM biotypes, but present in the three monocot PM genomes, indicating remarkable, independent and perhaps ongoing gene loss in different PM lineages. Consistent with this, we found that only 4192 (3819 singleton) genes are shared by all the eight PM genomes, the remaining genes are lineage- or biotype-specific. Strikingly, whereas the three monocot PM genomes possess up to 661 genes encoding candidate secreted effector proteins (CSEPs) with families containing up to 38 members, all the five dicot PM fungi have only 116–175 genes encoding CSEPs with limited gene amplification.

**Conclusions:**

Compared to monocot (grass) PM fungi, dicot PM fungi have a much smaller effectorome. This is consistent with their contrasting modes of host-adaption: while the monocot PM fungi show a high-level of host specialization, which may reflect an advanced host-pathogen arms race, the dicot PM fungi tend to practice polyphagy, which might have lessened selective pressure for escalating an with a particular host.

**Electronic supplementary material:**

The online version of this article (10.1186/s12864-018-5069-z) contains supplementary material, which is available to authorized users.

## Background

Powdery mildew is one of the most important and widespread plant diseases caused by ascomycete fungi belonging to the order of Erysiphales. Over 10,000 plant species are susceptible to powdery mildew (PM) diseases. These include staple crops such as wheat and barley, and important horticultural crops such as tomato, grapevine and strawberry [1, 2]. PM fungi are obligate biotrophic pathogens that strictly require living host cells to complete their life cycle. Like rust fungi and oomycete pathogens, PM fungi differentiate appressoria that penetrate the host plant cell wall and further develop feeding structures (i.e. haustoria) to extract nutrients and water from host cells [3, 4]. Interestingly, while monocot PM fungi display narrow host ranges [e.g. *Blumeria graminis* ‘*formae speciales*’ *tritici* and *hordei* can infect only wheat or barley, respectively [5]], some dicot PM fungi are capable of infecting hosts belonging to different plant families [6]. For example, *Oidium neolycopersici* is capable of infecting plants from 13 families including Arabidopsis and tomato that have diverged > 100 million years ago [7, 8]. How obligate biotrophic fungi adapt to a single or numerous host species remains to be an important open question.

Because powdery mildew fungi cannot be cultured in vitro and there is no effective method for genetic transformation, it is very difficult to conduct functional studies of PM fungi via conventional genetic manipulations. The next generation sequencing (NGS) and other OMICS technologies have been used to understand the genome evolution of PM fungi in relation to host-adaption [9]. Such studies however have mainly been focused on PM fungi infecting monocot plants belonging to the subfamily Pooideae of Poaceae, the only taxon of monocot plants that can be infected by PM fungi. These grass PM fungi are considered to have originated from a single species *Blumeria graminis* [10] and a *forma specialis* (f.sp.) is used to describe a *B. graminis* form specialized on a particular host species. The genome of *B. graminis* f.sp. *hodei* (*Bgh*) specialized on barley was first sequenced, partially assembled, annotated and analyzed [11], which was followed by similar work with *B. graminis* f.sp. *tritici* (*Bgt*) specialized on wheat [12]. A recent study further improved the genome assembly of two *Bgh* isolates, DH14 and RACE1, to a near-chromosome level, which has led to the annotation of 7118 genes for DH14 and 7239 for RACE1 [13]. These studies revealed that the three *B. graminis* genomes (two *Bgh* and one *Bgt*) are large in size (120-180 Mb) yet contain surprisingly few protein-coding genes (~ 7000 genes in total) compared with closely related necrotrophic ascomycete fungi such as *Botrytis cinerea* and *Sclerotinia sclerotiorum* (11,707 and 10,175 from EnsembleFungi Database) [14–16]. Both the extraordinary genome expansion and gene loss are associated with massive retrotransposon proliferation in these PM genomes, which is believed to be consistent with the obligate biotrophic parasitic style of the pathogens [11]. Remarkably, despite a low gene content, ~ 8% of the total annotated genes in these two genomes encode candidate secrete effector proteins (CSEPs) that are presumed to increase virulence on specific hosts [9, 11, 12]. More recently, Menardo and colleagues sequenced the genomes of several other grass PM fungi and conducted comparative genome analysis to reconstruct the evolutionary history of different *B. graminis* lineages. The authors found that in most cases, different *B. graminis formae speciales* (ff.spp.) fungi have co-evolved with their hosts, leading to host specialization. Yet, there are exceptions where possible host jumps or host range expansions might have occurred [17]. Additionally, while the majority of grass PM fungi are monophylogenetic, there may be exceptions; for example, there are different *B. graminis* isolates affecting *Dactylis glomerata*, and therefore *B. g.* f.sp. *dactylidis* is probably not a monophyletic group [17, 18]. More interestingly, Menardo and colleagues also discovered that *B.g.* f.sp. *triticale* capable of infecting the artificial hybrid crop triticale originated through a hybridization of isolates of the *Bgt* and of the *B.g.* f. sp. *secalis* [19]. These findings suggest that multiple mechanisms have contributed to the genome evolution of *B. graminis* ff.spp., which is believed to reflect their co-evolutionary struggle with their respective host plants.

Previous extensive studies demonstrate that barley and wheat resistance (*R*) gene conferring race-specific resistance to isolates of *Bgh* or *Bgt* encode immune receptors belonging to the nucleotide-binding leucine-rich repeat (NB-LRR) superfamily and that multiple *R* alleles exist at the barley *Mla* locus [20, 21] and the wheat *Pm3* locus [22–24]. The availability of *B. graminis* genome and transcriptome data have accelerated the identification of the Avirulence (*Avr*) genes that are recognized by some of the characterized *R* genes. Not surprisingly, several recently identified PM *Avr* genes that are recognized by alleles of *R* genes at the barley *Mla* locus, or the wheat *Pm3* or the *Pm2* locus encode canonical CSEPs [25–27]. Moreover, a recent comparative genomic analysis of several host-specific *B. graminis* lineages found evidence for a rapid turnover of *CSEP* genes as well as lineage-specific (LS) amplification of several *CSEP*s through repeated gene duplication [13, 28]. This analysis along with other studies also identified typical signatures of positive selection on effector genes of different *B. graminis* lineages presumably due to host-imposed selection pressure [12, 28, 29]. Thus, genetic, genomic and molecular evidence support a notion that there has been an arms race between *B. graminis* ff.spp. and their monocot hosts especially after domestication [28, 30, 31].

Compared to the extensive genomic studies of monocot PM fungi, there have been only limited whole-genome scale studies on dicot PM fungi [9]. Spanu et al. compared the *Bgh* genome sequence with those of two dicot PM biotypes, *Erysiphe pisi* (*Ep*) infecting pea, and *Golovinomyces orontii* (*Go*) infecting Arabidopsis, and showed that only seven and four *Bgh* CSEPs have identified homologous CSEPs in *E. pisi* and *G. orontii*, respectively [11]. This result suggests that the effectoromes of the monocot PM and dicot PM are highly divergent. However, the sequence coverage for these two dicot PM genomes was considered to be too low (~8X) for building high quality scaffolds for gene annotation [11]. A transcriptome analysis of enriched Arabidopsis PM *Golovinomyces orontii* haustoria revealed only 70 expressed CSEPs [32]. Interestingly, despite having a similar genome size (~ 126 Mb) as *Bgh*, the genome of *E. necator* (a PM infecting grapevine) contains only ~ 150 CSEP genes, further suggesting that dicot PM fungi may be significantly different from monocot PM fungi in terms of effector gene evolution. Apparently, high-quality genome sequences from additional dicot PM fungi are needed to allow identification and generalization of genome-scale sequence patterns (for effector genes in particular) that may reflect distinct modes of host adaption/specialization of different PM fungi.

In the current study, we obtained and assembled the whole genome sequences of four dicot PM fungi with distinct yet overlapping host ranges*.* Intriguingly, we found that the tomato PM genome is much smaller than the other three dicot PM genomes despite that it contains a similar number of genes. By comparing the predicted genes of the four dicot PM fungi to those of *Bgh* (both DH14 and RACE1) [13], *Bgt* and *E. necator,* we were able to identify genes that are conserved in all eight PM fungi genomes, or present in all monocot or all dicot PM, or specific to a particular PM lineage or PM biotype. Interestingly, compared to the predicted effectoromes of *Bgh* and *Bgt*, the size of the predicted effectoromes of all five dicot PM fungi is much smaller, so is the level of amplification of particular effector gene families. This genomic feature probably reflects a relatively lower level of the arms race between the dicot PM fungi and their host plants, which may be partially attributable to the polyphagous nature of these dicot PM fungi.

## Results

### Identification of three dicot powdery mildew biotypes and their infection tests on five host plant species

**Fig. 1:**
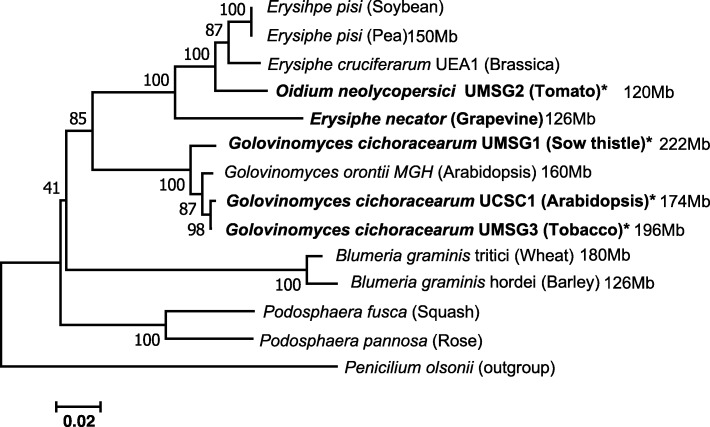
An ITS sequence-based phylogenetic tree of 13 powdery mildew pathogens including the four biotypes (indicated by *) used in this study was constructed using Mega 7.0 based on the Neighbor-Joining method. Percent bootstrap support values on the basis of 1000 replicates are shown next to the branches. The scale bar indicates average number of nucleotide substitutions per site. The estimated genome size known from literature or this study is indicated for nine PM biotypes. The ITS sequences were obtained in this study or retrieved from Genbank with the following Accession numbers matching the sequences from top to bottom of the phylogenetic tree: AF011306, FJ378879, AF031283, KX776199, KJ539202, HM449077, AF009176, AF031282, KR611314, KJ184337, HM484333, AF011321, AF011322, EF634440

To explore the genome features of dicot PM fungi in relation to host adaptation, we identified and purified three new dicot PM fungi over the past few years from the surroundings of the University of Maryland Shady Grove campus (UMSG) at Rockville, Maryland. They are sow thistle PM (*Golovinomyces cichoracearum* (*Gc*) UMSG1 (*Gc*M1)) [33], tomato PM (*Oidium neolycopersici* (*On*) UMSG2 (*On*M2)) and tobacco PM (*Gc* UMSG3 (*Gc*M3)). We assigned them to respective PM biotypes (with a unique UMSG-tag) according to their phylogenetic relationships based on the internal transcribed spacer (ITS) of the rDNA sequences (Fig. 1), which was later validated by phylogenetic analysis based on highly conserved single copy orthologous gene clusters existing in all the eight PM genomes (Additional file 1: Figure S1, see later section).

We then tested the host ranges of these three new PM biotypes along with the *Gc* UCSC1 (*Gc*C1) isolate [34] that has been widely used by the Arabidopsis community on five plant species belonging to four different plant families: Arabidopsis (*A. thaliana*, Col-0), cucumber (*Cucumis sativus*, straight eight), tomato (*Solanum lycopersicum,* Moneymaker), tobacco (*Nicotiana benthamiana*), and sow thistle (*Sonchus oleraceus*). Interestingly, we found that except for *Gc*M1, which is only infectious on sow thistle, the remaining three biotypes could complete their life cycle in at least two different hosts, with the tomato PM (*On*M2) being infectious on four of the five host plants except sow thistle (Table 1, Additional file 1: Figure S2-S4; [33]). Furthermore, all the four PM biotypes have evolved the ability to overcome penetration resistance in wild-type Arabidopsis accession Col-0 [33] and, except for *Gc*M1, could sporulate profusely on immune-compromised mutants such as *eds1–2* (Additional file 1: Figure S2-S4) and *pad4–1*/*sid2–1* (Table 1), indicating that these dicot PM fungi are likely polyphagous and have retained or evolved mechanisms that enable them to overcome penetration resistance and post-penetration resistance of multiple plant species including Arabidopsis. The polyphagous nature of these dicot PM fungi is in sharp contrast to the high host specificity of *Bgh* and *Bgt*. Hence, an interesting question is why host adaptation of dicot PM fungi is so different from that of monocot PM fungi?

**Table 1:** Characterization of host-ranges of four dicot powdery mildew biotypes^a^

Biotypes^b^	Tomato	Cucumber	Tobacco	Arabidopsis	Sow thistle	Arabidopsis *pad4–1/sid2–1*
*Gc*C1	–	++++	++	++++	–	++++
*Gc*M1	–	+/−	–	+/−	++++	++++
*On*M2	++++	+++	+++	++	–	++++
*Gc*M3	–	–	++++	+	–	++++

^a^The infection phenotypes were assessed visually or with the aid of a microscope. “-”, no or poor germination of conidia without successful penetration; “+/−”, successful penetration but without sporulation; “+”, poor sporulation with HR; “++”, moderate sporulation with no or weak HR; “+++”, good sporulation; “++++”, profuse sporulation

^b^*Gc*C1, *Golovinomyces cichoracearum* UCSC1; *Gc*M1, *G. cichoracearum* UMSG1; *On*M2, *Oidium neolycopersici* UMSG2; *Gc*M3, *G. cichoracearum* UMSG3

### The tomato powdery mildew has a relatively more compact genome

To understand genomic features of dicot PM fungi, we sequenced the four dicot PM biotypes using Illumina high throughput sequencing. We generated more than 80 million high-quality short reads (100 bases pair-end) for each biotype. We then performed de novo genome assembly using CLC Bio Genomic Workbench (v11) (http://www.clcbio.com/products/clc-genomics-workbench/). Interestingly, the total assembled genome size of the four PM fungi varies from 45 Mb (*On*M2) to 71 Mb (*Gc*M1) (Table 2).

**Table 2:** Genome sequence analysis and gene prediction of four dicot powdery mildew biotypes

PM Biotype	*Gc*C1	*Gc*M1	*Gc*M2	*Gc*M3
Raw Reads	84,214,356	89,713,850	86,621,454	82,892,794
Trimmed Reads	81,469,810	85,393,708	84,044,588	78,373,242
Scaffolds	22,821	25,102	14,365	22,581
N50 scaffold length	4625	4846	6751	4696
% Scaffolds	33.3	35	35.6	32
% Genome	39.8	44	30.1	38
Conitgs	30,165	33,822	18,861	29,563
N50 contig length	4095	4312	5946	4253
CEGMA^a^	99% / 99%	99% / 99%	99% / 99%	99% / 99%
Assembly Size (Mb)	64.64	71.27	45.08	65.02
Genome size^b^ (Mb)	173.8	221.8	120	195.8
Sequencing depth	50×	46×	73×	44×
Gene number	6718	6620	6961	6865
SP^c^ gene number	472	478	499	489
CSEP^d^ gene number	159	163	175	174

^a^CEGMA: Core Eukaryotic Genes Mapping Approach. Two hundred forty-eight core eukaryotic genes are used to evaluate the completeness of each scaffold

^b^Genome size is estimated by *K*-mer frequency

^c^SP: Secreted proteins without transmembrane domain

^d^CSEP: Candidate secreted effector proteins without homologs outside powdery mildew fungi

To evaluate the quality and completeness of the assembly, we conducted CEGMA (core eukaryotic genes mapping approach) analysis [35]. The CEGMA completeness is ~ 99%, which is in agreement with the BUSCO analysis (See Methods), indicating a high quality of our assembly. The CEGMA results show that only one of the 248 core eukaryotic genes (*CEGs*), i.e. *KOG2531* encoding xylulose kinase, is completely missing. Another *CEG*, *KOG0894* encoding an ubiquitin-conjugating enzyme E2, is partially deleted in all of the four genomes. The (partial) absence of these two genes is unlikely due to inadequate sequencing or sequence analysis, because (i) our genome/transcriptome sequences are of high quality; (ii) the four PM biotypes (this study; Table 2) and the grapevine PM *E. necator* [36] share the same characteristics; and (iii) KOG0894 is also partially deleted in monocot PM fungi. Interestingly, *KOG2531* is present in the genomes of monocot PM. Thus, loss of *KOG2531* in dicot PM fungi might have occurred independently after the divergence of dicot and monocot PM species.

Next, we estimated the genome size of these four PM biotypes based on *k*-mer counts [37]. We found that *Gc*C1, *Gc*M1, and *Gc*M3 have an estimated genome size of 173.8–221.8 Mb (Table 2), which is close to that of *Bgt* (~ 180 Mb) [12] and also the other two dicot PM - *G. orontii* (160 Mb) and *E. pisi* (150 Mb) [11]. However, *On*M2 has an estimated genome size of around 120 Mb, which is notably smaller, yet comparable, to that of *E. necator* C-strain (*En*C) or *Bgh* (~ 126 Mb) [11, 36]. Despite much effort, only 32.1–37.6% of the four genomic sequences could be assembled into scaffolds (Table 2). Because unassembled sequences are more likely to be highly repetitive, this result is consistent with the notion that PM genomes contain mostly repetitive sequences [11, 12, 30]. Further analysis showed that transposable elements (TE) account for 54.7%, 59.8%, and 51.9% of the assembled sequences in *Gc*C1, *Gc*M1, and *Gc*M3, respectively, whereas TE sequences only account for 39.4% in the case of *On*M2 (Additional file 2: Table S1). This result is consistent with the above results on genome size estimation and suggests that the *Golovinomyces* lineage might have undergone higher levels of TE proliferation compared to *On*M2.

We then examined genes likely involved in controlling proliferation of repetitive sequences. Repeat Induced Point Mutation (RIP) is a homology-dependent gene silencing process [38] that is believed to provide a defense against the spread of TEs and limit generation of paralogous genes in fungi [39]. Like the other PM fungi [11, 12], these four dicot PM fungi all lack genes involved in RIP. In addition, there is no significant difference between these four dicot PM fungi and the previous three PM fungi in possession of genes known to be involved in other TE-controlling mechanisms, such as quelling [40] and meiotic silencing of unpaired DNA [41] (Additional file 2: Table S2). Therefore, it is possible that the *Golovinomyces* lineage may have either lost an unknown gene(s) that limits TE proliferation or evolved a gene(s) that promotes TE proliferation.

### Genome annotation of the four dicot powdery mildew biotypes

To see how the four dicot PM genomes may distinguish from each other and how dicot PM genomes differ from monocot PM genomes in gene composition, we first performed ab initio genome annotation. The number of genes that could be reliably predicted by MAKER (v2.31.8) [42] was ~ 4000 for each of the four PM genomes (data not shown). We thus generated gene expression data from mycelia of the four biotypes using RNA-seq to improve gene prediction. By combining gene expression-based evidence and protein sequence homology-based evidence with results of ab initio analysis, we identified 6718, 6620, 6961, 6865 putative protein-coding genes for *Gc*C1, *Gc*M1, *Gc*M2 and *Gc*M3, respectively, in assembled scaffolds (Table 2), which is very close to the predicted number of protein-coding genes in other sequenced PM genomes [11–13, 36]. To evaluate the overall gene conservation of PM fungi as a group of ascomycete fungi in the order of Erysiphales, we did BLASTP search for all the genes encoded by the genomes of the four dicot PM in this study, and those encoded by the genomes of *E. necator*, *Bgh* (including isolate DH14 (version 4) designated *Bgh*D and isolate RACE1 designated *Bgh*R1) [13], and *Bgt* against genes encoded by other fungal genomes. We found that 39.0–40.3% of the total genes in each PM genome have homologs in yeast (*Saccharomyces cerevisiae* S288C), and 71.5–73.7% have homologs in the closely related fungi *Botrytis cinerea* (e-value< 10^− 10^, identity > 0.3, hit coverage > 0.5) (Additional file 2: Table S3). Additionally, we found that ~ 82% of the predicted genes from the four PM genomes have homologs in the Ensemble Fungi database (which contains 802 genomes except those of PM fungi with 7,461,030 protein coding genes) based on BLASTP search results (e-value< 10^− 10^, identity > 0.3, hit coverage > 0.5). While this result further validates the high quality of our sequences and gene prediction, it also indicates that the majority of PM genes are probably structural genes and functionally conserved.

### Gene losses in powdery mildew genomes

**Fig. 2:**
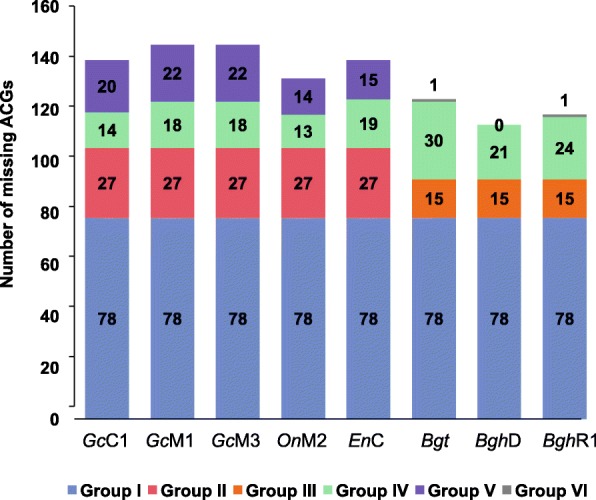
Number of Ascomycete core genes (ACGs)missing in each PM biotype. Group I: those missing in all ten PM fungi; Group II: those missing in all dicot PM fungi but present in all monocot PM fungi; Group III: those missing in all monocot PM fungi but present in all dicot PM fungi; Group IV: 30 ACGs missing in both dicot and monocot PM fungi but not all. Group V: 35 ACGs missing in some dicot PM fungi but present in all monocot PM fungi; Group VI: 1 ACGs missing in some monocot PM fungi but present in all dicot PM fungi; *Gc*C1: *Golovinomyces cichoracearum* UCSC1; *Gc*M1: *G. cichoracearum* UMSG1; *On*M2: *Oidium neolycopersici* UMSG2; *Gc*M3: *G. cichoracearum* UMSG3; *En*C: *Erysiphe necator* C-strain; *Bgh*D: *Blumeria graminis* f.sp. *hordei* DH14; *Bgh*R1: *Blumeria graminis* f.sp*. hordei* RACE1; *Bgt: B. g.* f.sp. *tritici*; Detailed information is provided in Additional file 2: Table S4

It is known that barley and wheat PM fungi have 6525–7239 genes, which is slightly more than half of the genes that are normally present in closely related ascomycete fungi such as *Botrytis cinerea* (11,707 genes) and *Magnaporthe oryzae* (12,593 genes) [11, 12, 14, 36, 43, 44]. However, it appears that PM fungi tend to keep ~ 93% (i.e. 3097) of the ascomycete core genes (ACGs) (i.e. 3328) shared by *B. cinerea*, *M. oryzae, Colletotrichum higginsianum, Sclerotinia sclerotiorum* and *S. cerevisiae*, with 186 ACGs missing in at least two of the eight PM genomes (TBLASTN e-value < 10^− 6^) [11, 36]. We divided these 186 ACGs into six groups based on their absence among the eight PM genomes to get an idea about the gene-loss process in PM fungi. As shown in Fig. 2 and Additional file 2: Table S4, Group I contains 78 ACGs missing in all eight PM genomes. This suggests that these genes were lost in an ancestral PM before the divergence between the monocot and dicot PM lineages. Interestingly, 60 of the group I ACGs are also absent in the non-Ascomycete biotrophic fungus *Puccinia graminis*. The apparent convergent loss of these ACGs in both Ascomyete and Basidiomycete fungi suggests that they are dispensable for biotrophic life of fungi. By contrast, 47 of the group I ACGs can be found in at least one obligate biotrophic fungus other than PM, implying that the loss of these ACGs most likely occurred in an ancestral PM genome after its separation from non-PM fungal lineages. Group II contains 27 ACGs that are missing in all the five dicot PM fungi but present in the three monocot PM fungi, while group III contains 15 ACGs that are only absent from all the three monocot PM genomes. These differential gene losses may contribute to the irreversible divergence between the two PM lineages in adaptation to dicot or monocot hosts, respectively. Finally, each of the 66 ACGs in the remaining three groups (IV, V & VI) is missing in at least one but not all of the eight PM fungi (for details, see Fig. 2). Intriguingly, it appears that the dicot PM fungi have lost more ACGs (i.e. 132–145) than the monocot PM fungi (i.e. 114–124). This is unexpected given that monocot PM fungi are considered to have evolved to a more advanced state of host-specialization whereas dicot PM fungi are more primitive in host adaptation with a broader host range in general [45]. Taken together, our above results indicate that significant gene loss had occurred in an ancestral PM before the monocot-dicot PM division, and that gene loss has been an ongoing process in the PM fungi. These results also suggest that lineage- or genome-specific gene loss may contribute to host-specialization of PM fungi and/or reflect the impact of host metabolisms on respective PM pathogens.

### Conservation and diversification of powdery mildew genes

**Fig. 3:**
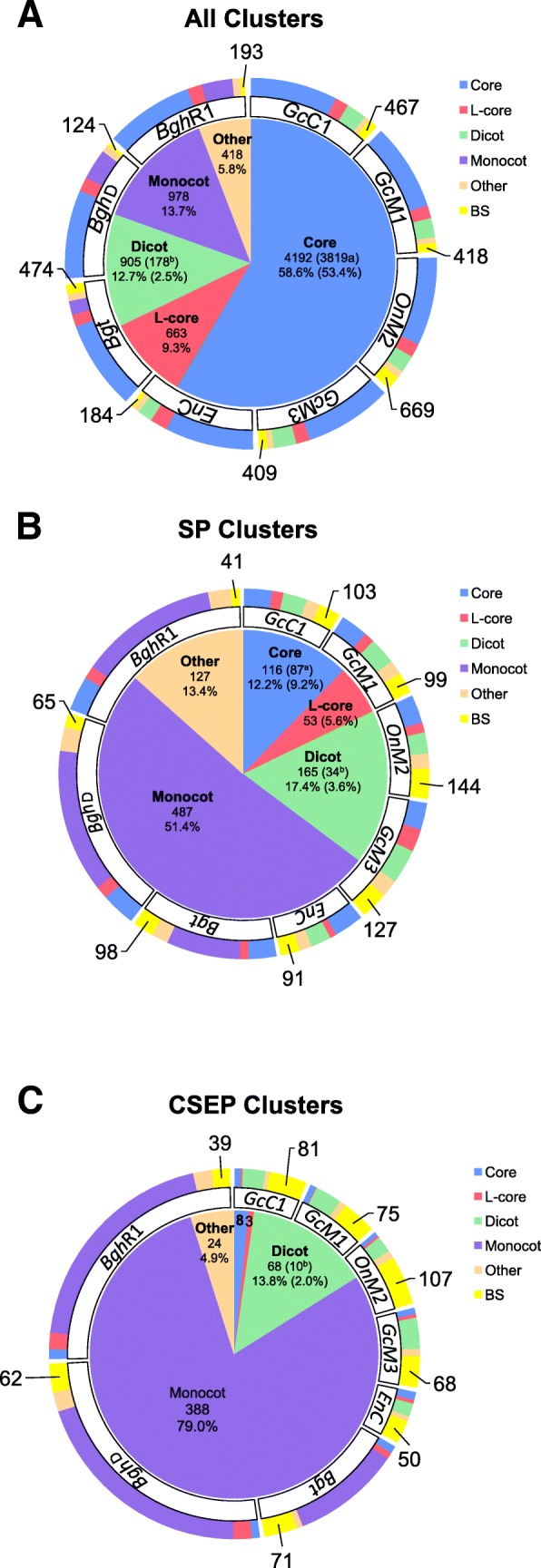
Identification of total gene clusters (**a**), secreted protein (SP) gene clusters (**b**) and candidate secreted effector protein (CSEP) gene clusters (**c**) across eight PM genomes. The central pie charts represent the proportion of gene clusters with different conservations; the peripheral circles represent the proportion of genes belonging to different clusters in each biotypes. The numbers outside the circle represent the BS genes for the eight indicated PM fungi (for details see Additional file 2: Tables S5 to S7). Core: gene clusters with members from all of the eight PM genomes (^a^ represents clusters containing only one members from each of the eight PM genome). L-core: gene clusters with members from seven of the eight PM genomes, which are likely core clusters. Dicot: gene clusters with members from only the dicot PM genomes (^b^ represents clusters containing members from all of the five dicot PM genomes). Monocot: gene clusters with members from only the monocot PM genomes. Other: gene clusters with member from both monocot and dicot PM fungi, but not all eight PM fungi. BS: biotype-specific genes which can not group with other genes or can only group with genes from its own genome. *Gc*C1: *Golovinomyces cichoracearum* UCSC1; *Gc*M1: *G. cichoracearum* UMSG1; *On*M2: *Oidium neolycopersici* UMSG2; *Gc*M3: *G. cichoracearum* UMSG3; *En*C: *Erysiphe necator* C-strain; *Bgh*D: *Blumeria graminis* f.sp. *Hordei* DH14; *Bgh*R1: *Blumeria graminis* f.sp. *Hordei* RACE1; *Bgt: B. graminis* f.sp. *tritici*

**Fig. 4:**
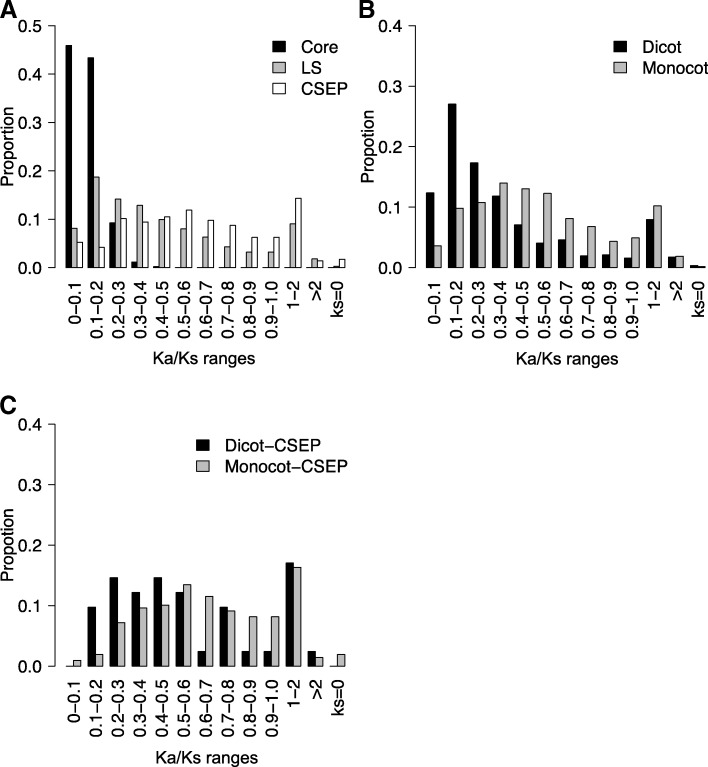
Calculation and frequency distribution of the ratio of the non-synonymous substitution rate (Ka) to the synonymous substitution rate (Ks) for genes in the specified categories. **a** A comparison among core gene clusters present in all the eight PM fungi (Core) with lineage-specific clusters (LS) and candidate secreted effector protein clusters (CSEP). **b** A comparison between dicot and monocot PM genes. **c** A comparison between dicot PM-specific CSEP clusters and monocot PM-specific CSEP clusters. The x-axis are Ka/Ks ranges

All PM fungi adopt the same invasive strategy. That is: they acquire nutrients from host epidermal cells by haustoria and co-survive with their host cells. To define a core set of PM genes that enable this invasive strategy and identify LS genes that may distinguish dicot PM fungi from monocot PM fungi, we deployed the OrthoFinder software to cluster all PM proteins predicted from the eight genomes and conducted a detailed comparative analysis. Our results show that the 54,530 PM protein-encoding genes from the eight analyzed PM genomes can be grouped into 7156 gene clusters (i.e. orthologous/homologous gene groups across different PM species; Additional file 2: Table S5) with 2 to 495 members from at least two PM biotypes (or isolates in the case of *Bgh*), plus 2771 unique genes that cannot be clustered with genes from any other PM genome or clustered only in a single PM genome (Fig. 3a; Additional file 2: Table S6). Among these 7156 gene clusters, 4192 (58.6%) are core gene clusters containing 4319 to 4424 genes in each of the eight PM genomes (Fig. 3a; Additional file 2: Table S6). An additional 663 clusters are defined as likely-core (L-core) clusters (Fig. 3a), because they contain members from seven (including *Bgh*D and *Bgh*R1) of the eight PM genomes (implying that its absence from the PM biotypes other than *Bgh* may be due to inadequate sequence coverage or annotation, although biotype-specific (BS) gene loss cannot be excluded) and nearly 98% of them have homologs in non-PM fungal genomes in the NCBI NR database, which is very similar to the core clusters (Additional file 2: Table S7). Among these core gene clusters, 3819 are singleton with each cluster comprising one member from each one of the eight PM genomes (Fig. 3a). Thus, these 3819 genes are likely conserved genes that serve basic cellular functions for all PM fungi and perhaps also define PM as a distinct group of ascomycete fungal pathogens. Indeed, GO annotation showed that the major functional categories of these 3819 genes include transmembrane transport, in addition to other essential biological processes such as DNA replication, RNA processing, and protein translation (Additional file 2: Table S8). Most interestingly, 24 of the 3819 core singleton genes have no close homolog (i.e. sequence homology e-value < 10^− 10^ at the protein level) outside PM fungi, suggesting that they might have evolved in an ancestral PM fungus after its speciation from a more ancient, non-PM fungus and/or these genes have been evolving faster than the other core genes. By contrast, there are 905 dicot PM-specific and 978 monocot PM-specific clusters (Fig. 3a), and 43.7% and 74.2% of them do not find homologs in other fungal genomes, respectively (Additional file 2: Table S7). This suggests that LS genes in general have evolved (or been evolving) very fast and that their evolution and maintenance may have contributed to the adaptation of the respective PM fungi to dicot or monocot hosts. To assess if LS genes have been under higher selection pressure imposed by their respective hosts, we calculated the ratio of nonsynonymous substitution rate (Ka) to synonymous substitution rate (Ks) for the LS genes as well as the core genes. We found that the Ka/Ks ratios for the LS genes (with a median value of 0.36) are significantly higher (Wilcoxon test *P*-value < 0.001) than those of the core genes (median = 0.11) (Fig. 4a). This indicates that the LS genes are more likely involved in host adaptation processes therefore under positive selection or relaxed purifying selection. Notably, the monocot PM-specific genes not only contain a relatively higher proportion (74.2%) of novel genes but also have higher Ka/Ks ratios (median = 0.49) when compared with the dicot PM-specific genes (43.7%; median = 0.25) (Fig. 4b), indicating that monocot PM fungi are probably under higher selection pressure from their specific monocot hosts.

In order to identify the types of genes enriched in the LS genes clusters, we performed InterProScan 5 [46] and Blast2GO [47] for functional annotation. We found that genes with unknown function are significantly enriched (77.6%) in LS clusters, whereas only 12.8% of the core gene complement are genes with unknown function (*p*-value < 0.0001; Additional file 1: Figure S5) and more than 89.3% (2190 out of 2771) of the unique genes have no annotated functions. Besides, 59.5% and 79.0% of the LS and BS genes have no homologs outside PM (Additional file 1: Figure S5), indicating that a large number of PM genes are novel and remain to be characterized. Based on the functional annotation of the remaining 1602 genes that belong to the LS clusters and have functional annotation, we found that genes in the monocot PM genomes are more significantly enriched in ribonuclease (RNase) activity and hydrolase activity, and also enriched in RNA polymerase II transcription factor activity (Additional file 1: Figure S6A). In comparison, despite that dicot PM-specific genes are also enriched in RNase and hydrolase activity, the degree of enrichment is not as significant as those of the monocot PM fungi (Additional file 1: Figure S6B). In addition, the dicot PM-specific genes are enriched in other functions, such as oxidoreductase activity, cation binding activity, and protein dimerization activity (Additional file 1: Figure S6B). Such differential functional enrichment for monocot and dicot PM genes likely underscores their distinct host adaptation mechanisms, and reflects the impact of different host physiology and immunity on the invading PM fungi during the long time co-evolutionary struggle with their respective hosts.

### Identification of gene categories associated with pathogenicity

To gain a better understanding of host-adaptation mechanisms of different PM fungi, we compared the differences in pathogenicity-associated genes. It is known that the monocot PM fungi have lost most of those genes encoding enzymes involved in production of secondary metabolites, which has been considered to be consistent with their obligate biotrophic life-style [11, 12]. Similar to what was reported for *Bgh*, *Bgt* and *En*C, only genes encoding two polyketide synthases (PKS) and one non-ribosomal peptide synthases (NRPS) are present in the genomes of the dicot PM biotypes we sequenced (Additional file 2: Table S9). On the other hand, it is known that fungal pathogens utilize a wide variety of carbohydrate-active enzymes (CAZy) in order to infect their host plants [48]. Consistent with this notion, we identified a total set of 124–135 CAZy genes predicted to encode 78 different types of catalytic modules (Additional file 2: Table S10). The number of modules in each type was nearly identical in all eight PM fungi.

**Fig. 5:**
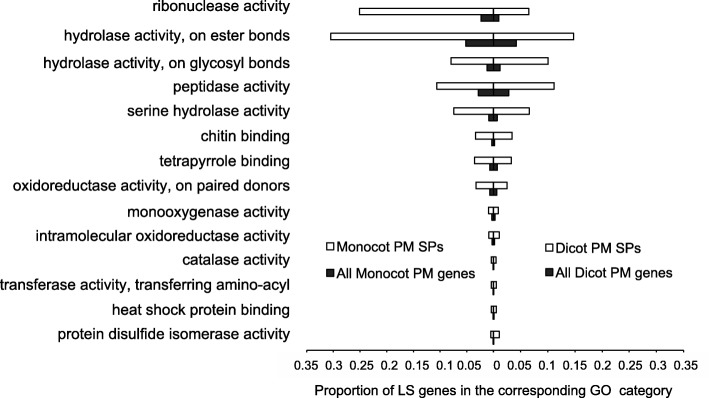
A Comparative Gene Ontology (GO) term enrichment analysis for dicot PM secreted protein genes (Dicot PM SPs) (Right) and monocot PM secreted protein genes (Monocot PM SPs) (Left). Fourteen categories of molecular functions which are significantly enriched in the secreted proteins with at least 2 fold change were selectively presented. The X-axis is the proportion of LS genes in the corresponding GO category

It is well established that secreted proteins (SPs) play essential roles in pathogenesis of bacterial and fungal pathogens. To define the secretomes of the four dicot PM fungi, we used SignalP3.0 to predict potential N-terminal secretion signal peptides and TMHMM 2.0 to predict transmembrane domains in the mature peptides. In total, we identified 472–499 potential SPs in the four PM biotypes (Additional file 2: Tables S6 and Table S11). The size of the four secretomes is similar to that of *En*C (422 SP-encoding genes without a transmembrane domain) but significantly smaller than that of *Bgh* (1090), *Bgh*R1 (1039), and *Bgt* (706) (Note, these numbers were derived by using the same criteria in this study; Additional file 2: Table S6). About 50% of the dicot PM SPs have annotated functions, whereas only ~ 35% of monocot PM SPs have annotated functions. Further GO term enrichment analysis showed that most of the molecular functions enriched in the SPs are similar between monocot and dicot PM fungi. These include RNase, hydrolase, peptidase, transferase, and oxidoreductase. However, one noticeable difference is that SPs of monocot PM fungi are far more enriched for RNase and hydrolase activities compared to the SPs of dicot PM fungi (Fig. 5). This is consistent with the findings that *Bgh* has evolved a superfamily of RNase-like effectors that are delivered into host tissues/cells to suppress host immunity [29] and hence also become targets of host immunity [49] (see more details in the later text).

To find out the size of effectoromes of the four dicot PM fungi, we tried to identify candidate secreted effector proteins (CSEPs) that are defined as those secreted proteins that lack any transmembrane domain and often lack homologs outside PM [11]. Based on these criteria, we identified 159, 163, 175, 174 CSEPs in the four dicot PM biotypes, which are much fewer than those in *Bgh*D (661), *Bgh*R1 (629) [29, 50] and *Bgt* (353/428) (Note 428 for *Bgt* is an adjusted number after considering genes highly homologous to *Bgh* effectors but without a canonical signal peptide, possibly due to incomplete sequence processing or inadequate annotation [12] (Additional file 2: Tables S6 and S11). Given that the grapevine PM (*En*C) genome contains even fewer CSEPs (116) based on our analysis, it appears that all the five dicot PM fungi have a small effectorome whereas both *Bgh* and *Bgt* possess a much expanded effectorome. This difference in effectorome size between dicot and monocot PM fungi is most striking, and it probably indicates a relatively lower level of the arms race between dicot PM and their hosts when compared with that between monocot PM and their hosts. This inference would agree well with the scenario in which polyphagous dicot PM fungi likely face relaxed selection pressure from any one particular dicot host.

To examine if there are a core set or LS SPs or CSEPs of PM fungi that may respectively contribute to the basic (haustorium-dependent and host epidermal cell-based) biotrophic life style or adaptation to specific hosts, we checked the conservation of orthogroup clusters of PM secretomes and effectoromes using OrthoFinder. As expected, the percentage of conserved core genes goes down dramatically in SPs, especially in CSEPs compared to the percentage based on the entire gene complement (Fig. 3b & c). Interestingly, we still found that 87 SP clusters including four CSEP clusters contain only one gene per cluster from each of the eight PM genomes. This suggests that these 87 genes encoding secreted proteins are conserved in all eight PM genomes and thus may play essential roles in PM pathogenesis (Fig. 3b & c; Additional file 2: Table S6). On the other hand, while 255 SP clusters including 199 CSEP clusters are shared by all the three monocot PM genomes while missing in the five dicot PM genomes, only 34 SP clusters including 10 CSEP clusters appear to be conserved in all five dicot PM genomes while missing in the three monocot PM genomes (Fig. 3b & c; Additional file 2: Table S6). Hence, these 34 SP proteins are likely important for PM adaptation to dicot hosts. Another interesting pattern we observed is that while there is an apparent amplification of many SP/CSEP gene families in the three monocot PM genomes (e.g. 114 SP clusters and 82 CSEP clusters have at least 2 members from *Bgh*D), there is a much lower level of effector gene amplification in the dicot PM genomes (e.g. only 21–27 SP clusters and 7–13 CSEP clusters have at least 2 members from any of the five dicot PM genomes) (Additional file 1: Figure S7). Again, these two contrasting patterns probably reflect different levels of the arms race between different PM lineages and their respective hosts. However, we can not exclude the possibility that many of the duplicated, highly similar dicot PM CSEPs may have collapsed in single sequences in our short read assembly.

It has been reported that the *Bgh* genome contains a superfamily of genes encoding RNase-like effectors [29] and several *Avr* genes recently characterized from *Bgh* and *Bgt* belong to this superfamily [25–27]. To see if the dicot PM genomes have similar amplification of such genes, we first identified all genes predicted to encode RNase-like proteins. As shown in Additional file 2: Table S12, there are 74–138 RNase-like genes in the five dicot PM genomes, which is similar to the gene numbers (89–138) found in the three monocot PM genomes. However, among those genes, only 6–14 are predicted to encode RNase-like effectors in the dicot PM genomes, which is far fewer than those (29 in *Bgt*, 79 *Bgh*D and 74 in *Bgh*R1) in the monocot PM genomes. Thus, unlike the monocot PM fungi (*Bgh* in particular), dicot PM fungi have only a limited amplification of genes encoding RNase-like CSEPs. Interestingly, two gene groups CSEP_OG0000001 (with 1–6 member from each isolate) and CSEP_OG0000002 (with 2–3 members from each isolate) contain RNase-like effector genes that exist in all the eight PM genomes, indicating that these RNase-like effector genes probably originated before the divergence of dicot PM and monocot PM fungi. Notably, a recent gene structure and phylogenetic analysis also suggested that those few functionally characterized *Avr* gene encoding RNase-like effectors in *Bgh* and *Bgt* probably originated from a single ancestral gene [49].

Previous studies identified sequence signature of positive selection in candidate effector genes of some pathogens including monocot PM fungi [12, 45]. To examine whether there is any difference in the signature of host-exerted positive selection between monocot and dicot PM fungi, we calculated the ratio of Ka/Ks for the CSEP gene clusters using PAML in comparison with those of the 3819 core gene clusters. The one-to-one core gene clusters have a Ka/Ks ratio less than 0.46, with an average of 0.11. In contrast, the CSEP genes have much higher Ka/Ks ratios, with an average of 0.66 (Fig. 4a), indicating that these PM CSEP genes are in general under higher level of positive selection or relaxed purifying selection. Interestingly, when the percentages of CSEP genes with differential Ka/Ks ratios were calculated (Fig. 4c), we found that close to 50% of the dicot PM CSEP genes have a Ka/Ks ratio < 0.5, whereas > 70% of the monocot PM CSEP genes have a Ka/Ks ratio > 0.5 with 41 having a Ka/Ks ratio > 1.0 (Fig. 4c). This indicates that monocot PM-specific CSEP genes in general are under higher selection pressure than the dicot PM CSEP genes. Intriguingly however, we also noticed that eight dicot PM CSEP genes have a Ka/Ks ratio > 1.0, indicating strong positive selection acting on these genes (Fig. 4c).

### Mycelium and Haustorium transcriptome analyses

The haustorium is the critical feeding structure of PM fungi. To identify PM genes that may be involved in haustorial functions (i.e. suppression of host immunity and acquisition of nutrients), we obtained both mycelial and haustorial transcriptome sequences from three of the four dicot PM fungi (*Gc*C1, *Gc*M3 and *On*M2). However, the haustorial transcriptome of *Gc*C1 was considered to be insufficient (only less than 0.9% of the total reads can be mapped to *Gc*C1 scaffolds) for further analysis (see Discussion for an explanation). While each of the mycelia RNA samples generated 32 M–102 M reads matching PM sequences, the RNA samples from haustorium-containing leaf tissues generated only 1.1 M–6.0 M reads that match PM sequences, accounting for only 1.3–3.8% of the total reads (Additional file 3: Table S13). The gene expression levels among different replicates are highly correlated (Additional file 1: Figures S8 & S9). With a FPKM (Fragments Per Kilobase of transcript per Million mapped reads) cutoff of 1.5 for reliable detection of gene expression, we found that on average, 82.4–94.4% of all PM genes are expressed in mycelia and/or haustoria of *On*M2 and *Gc*M3 (Additional file 3: Table S14), indicating that most genes are required for the asexual life cycle of powdery mildew.

We then set fold-change > 2 and q-value < 0.05 as the cut-off for differential expression (DE). We found that 16.1% (1118) and 27.4% (1884) of all the predicted genes are significantly differentially expressed in haustoria relative to mycelia for *On*M2 and *Gc*M3, respectively (Table 3). Among these DE genes, 42.6% and 30.9% have no annotated functions, suggesting that genes involved in haustorial biogenesis and functioning are poorly understood and/or haustorium-related DE genes are fast evolving. GO enrichment analysis of the genes with known function revealed that the up-regulated DE genes are enriched in ribonuclease activity, oxidoreductase activity and hydrolase binding, etc., while the down-regulated DE genes are enriched in transferase activity and signaling receptor activity, etc. (Additional file 3: Tables S15 & S16). Notably, four up-regulated genes from *On*M2 are predicted to encode three sugar transporters and one amino acid transporter, while six such genes from *Gc*M3 encode five sugar transporters and one amino acid transporter. This implies that these transporter genes may play an important role in uptake of host nutrients in haustoria. Interestingly, the GO terms enriched in *On*M2 and *Gc*M3 are largely different (Additional file 3: Tables S15 & S16), suggesting that *On*M2 and *Gc*M3 differ greatly in gene regulation in haustorial cells. This is also supported by the observation that although ~ 75% of the total genes are shared by *On*M2 and *Gc*M3, only ~ 22.1% (198) of 894 up-regulated genes in *Gc*M3 are also up-regulated in *On*M2 (Additional file 3: Tables S17 & S18). Among the 198 genes that are up-regulated in haustoria of both *On*M2 and *Gc*M3, 83.3% (165) are core or core-like genes and only four are CSEPs. *Gc*M3 is most closely related to *Gc*C1 (they diverged ~ 1.3 MYA). To see if these two closely related PM fungi are more similar in gene regulation in haustorial cells, we compared up-regulated genes of *Gc*C1 in haustoria (this should be relatively reliable despite the overall representation of *Gc*C1 RNA is deemed insufficient for comparison). We found that 56.3% (503) of the 894 up-regulated genes (of which 39 are CSEPs) in *Gc*M3 haustoria are also up-regulated in *Gc*C1 haustoria (Table 3; Additional file 3: Tables S17 & S19). This result suggests that gene regulation mechanisms in haustorial cells have undergone a much higher level of diversification between different PM tribes (*Golovinomyces* vs. *Oidium*) compared to that within a tribe (*Gc*C1 vs. *Gc*M3).

**Table 3:** Number of genes differentially expressed in haustoria relative to mycelia in *On*M2 and *Gc*M3

Category	*On*M2			*Gc*M3		
	All	SP	CSEP	All	SP	CSEP
Up	644	86	47	894	128	65
	9.25%	17.23%	26.86%	13.02%	26.18%	37.36%
Down	474	68	22	990	112	22
	6.81%	13.63%	12.57%	14.42%	22.90%	12.64%
Total	1118	154	69	1884	240	87
	16.06%	30.86%	39.43%	27.44%	49.08%	50.00%

Genes with expression fold change (haustoria/mycelia) ≥2 and q-value < 0.05 are considered as differentially expressed

Most notable is that 69 (47 up) CSEPs of *On*M2 and 87 (65 up) CSEPs of *Gc*M3 which respectively account for 39.4% (26.9% up) and 50.0% (37.4% up) of their total CSEPs were found to be differentially expressed (Table 3). However, the percentages of total genes showing differential expression in haustoria for *On*M2 and *Gc*M3 are only 16.1% (9.3% up) and 27.4% (13.0% up), respectively (Table 3). These results indicate that a higher proportion of effector genes are selectively up-regulated in haustorial cells, agreeing with their anticipated functions in suppressing host defense and facilitating nutrient uptake. Those CSEP genes highly induced in haustoria more likely play important roles in PM pathogenesis *in planta*.

## Discussion

In this study, we sequenced four dicot PM biotypes that exhibit partially overlapping host ranges, analyzed and compared their genome sequences with three monocot PM genomes (*Bgh*D*, Bgh*R1 and *Bgt*) and the grapevine PM (*E. necator*) genome. Apart from the finding that PM genomes vary considerable in size (ranging from 120 to 222 Mb), we have revealed genes or gene families that are either conserved in all eight PM genomes, or are lineage- or biotype-specific, and identified candidate effector genes or gene families that may underscore the biological differences in relation to host adaptation displayed between these PM fungi.

### Gene count: How many genes in PM genomes?

All the eight PM genomes sequenced have 6525–7239 predicted genes dispersed in ~ 120 Mb to ~ 222 Mb DNA sequences (Table 2), indicating low gene density (3–6 gene/100 Kb) for PM fungi. The small size of assembled scaffolds for the four genomes (N50: 4095–5946 bp) is due to both the limitation of short-read sequencing strategy and the highly repetitive nature of the PM genomes (Table 2). Therefore, even though > 99% of the gene regions are covered based on the assessment using CEGMA, there may still be some gene models (314–497) that are truncated in de novo gene prediction and they are more likely to be present near the end of scaffolds (Additional file 1: Figure S10). However, through RNA-seq analysis, we found that 85% and 71% of those partial genes from *On*M2 and *Gc*M3 were expressed in haustoria and/or mycelia (Additional file 3: Table S20). Hence, we included all the partial gene models in our estimation of total number of genes. Among these partial genes, the ones with standard start codon (142–254) will not affect our effector prediction, since we can predict signal peptide and transmembrane domain of the N terminal protein sequences. The remaining 129–243 partial genes do not have a standard start codon and we cannot perform signal peptide prediction. In our study, we found that most of those effectors are lineage specific and have no homolog outside PM. However, only 68–111 of the 314–497 partial genes are lineage specific (implying that they may encode effectors). Consequently, we think even though there are 314–497 partial gene models that remain to be resolved in the future when the genomes are better assembled, this should not significantly affect our estimation for the total number of genes and effector genes.

Interestingly, among 6525–7239 genes in a given PM genome, 4192 core (of which 3819 are singleton clusters shared by all the eight PM genomes) and 663 likely core genes clusters (shared by seven PM genomes) are probably required for maintaining essential life processes conserved in all fungi, Thus, these conserved genes (accounting for ~ 75% of all genes in any PM biotype) represent the “core” PM genome content (Fig. 3a). On the other hand, there are 925–1722 LS and/or BS genes in each PM genome that may determine the characteristics of the invasive strategies and host-adaptation for each specific PM biotype (Additional file 2: Table S6). Thus, these genes (accounting for ~ 25% of all genes in any PM biotype) represent the “variable” PM genome content (Fig. 3a). Given that there are 24 core PM genes that are uniquely present in all examined PM fungi, it is tempting to speculate that these 24 genes may play essential roles in making PM fungi “powdery mildews”.

### Genome size variation in powdery mildew fungi

Taking the genome size information of *E. pisi* and *G. orontii* into consideration, it is apparent that there is a high-level of variation in genome size among the 10 sequenced PM genomes, which ranges from 120 Mb to 222 Mb. According to the published data, the *Bgt* genome is 54 Mb bigger than the *Bgh* genome, which is surprising given that they diverged only about 6.3 MYA [11, 12]. In this study, we also found remarkable genome size variation among different dicot PM fungi which range from 120 Mb (*On*M2) to 222 Mb (*Gc*M1). Despite that the total number of genes predicted in each of the eight sequenced PM genomes considered in this study is similar (between 6525 and 7239), there are only 4192 core PM genes identified among these PM genomes (Table 2), suggesting that more than 25% of the PM genes are variable. This notion is also reflected by the differential loss of some ACG genes in different PM lineages and/or biotypes (Fig. 2). Hence, we can make the following three generalizations with regard to genome evolution of PM fungi. First, PM genomes are relatively large (120 Mb to 220 Mb) and featured with a high level of plasticity compared to other Ascomycete fungi whose genome size ranges from 30 Mb to 60 Mb [16]. This genomic feature for PM genomes is likely attributable to differential proliferation of repetitive sequences in different PM genomes, which might be associated with the loss of RIP controlling TE proliferation in an ancestral PM. Second, the PM genome size per se does not seem to be associated with host specificity or adaption, implying that genome expansion in individual PM genomes might have occurred fortuitously and largely independently. Third, given that *Gc*M1 has the largest genome with a predicted effectorome much smaller than those of *Bgh* and *Bgt* (Table 2), one may infer that TE proliferation and the resultant genome size expansion alone without host-imposed selection pressure does not necessarily lead to increased generation of novel effector genes.

### Effectoromes: arsenals reflecting the levels of the host-pathogen arms race

Genome sequencing has revealed a large repertoire of candidate effector genes for many obligate biotrophic fungal and oomycete pathogens [51–53]. For example, the genomes of two monocot powdery mildew *Bgh* and *Bgt* contain a large number of CSEP genes accounting for 8% and 9% of the total genes, respectively [11, 29]. The evolution of large-sized effectoromes in these pathogens likely reflect a high level of arms race with their respective hosts. Indeed, extensive studies on the genetic/molecular basis of race-specific resistance in barley and wheat fully support this notion. First, there exists a high level of allelic diversity for the CC-NB-LRR–encoding *R* at the barley *Mla* locus [20, 21] and in the wheat *Pm3* locus [22–24] that confer race-specific resistance to PM fungi. Second, several PM *Avirulence (Avr)* genes that are recognized by their cognate *R* genes at the barley *Mla* locus, or the wheat *Pm3* or the *Pm2* locus indeed encode canonical CSEPs [25–27]. Moreover, a recent comparative genome analysis of the effectoromes of *Bgh* and *Bgt* and three other host-specific lineages of *B. graminis* found that there have been lineage-specific expansions of several clades of CSEP genes through repeated gene duplications and the effector repertoire of *B. graminis* is subjected to an extremely rapid turnover, likely as an expected outcome of the evolutionary host-pathogen “arms race” [28].

By contrast, despite great efforts, only a few dominant *R* genes for PM resistance in dicot plants have been characterized/isolated [54–56]. Among these dominant *R* genes conferring resistance to PM fungi in dicot plants, the best understood are two atypical *R* genes *RPW8.1* and *RPW8.2* from Arabidopsis [8]. Proteins encoded by these two *RPW8 R* genes are small basic proteins that are unlikely to function as receptors to activate ETI; rather they are targeted to the extrahaustorial membrane where they activate defense to constrain the haustorium, therefore conferring broad-spectrum resistance [57, 58]. In many cases, resistance to PM fungi in dicot plants is caused by natural loss-of-function mutations in *MLO* genes [59–64]. Curiously, to date, not a single dicot *NBS-LRR* gene has been definitively characterized to confer race-specific resistance to dicot PM fungi, many of which including *Gc*C1 and *On*M2 have a very broad host range [65, 66]. Therefore, one may speculate that the level of the arms race between PM fungi and their dicot hosts is rather primitive. Results from this study on PM genomes is compatible with this speculation: all five dicot PM contain a small-sized effectorome (116–175), which accounts for only 1.8–2.5% of the total number of genes in each of the five genomes (Additional file 2: Table S21). It is worth pointing out that our haustorial transcriptome data were derived from leaf tissues at 6 days post-inoculation (dpi) when haustoria with different ages from the original as well as the secondary host cell penetration events must be present in the infected leaf tissues. Thus, our haustorial transcriptome data should capture most of the haustorially expressed genes including effector genes. However, we cannot exclude the possibility that some effector genes are only expressed in primary haustoria formed in naïve plant tissues at earlier time points and such effector genes might have been missed. It is important to point out that the sheer larger size of the effectoromes of *Bgh* and *Bgt* is primarily due to the amplification of many (e.g. 82 in *Bgh*) CSEP families with up to 38 members per family, which may be considered to be a typical molecular signature of an escalated arms race [67, 68]. By contrast, all dicot PM genomes contain only 116–175 CSEPs with little amplification (< 8 genes per family) for a small number of CSEPs (especially in *En*C) or no amplification at all for most CSEP genes (Additional file 1: Figure S7). In addition, the dicot PM-specific genes including the dicot PM-specific CSEP genes have relatively lower Ka/Ks ratios when compared to those of the monocot PM genes, which agrees with the inference that a broad host range for dicot PM fungi would relax the selection pressure from any particular resistant host on the survival of the PM pathogens in the natural or even agricultural settings. However, it should be pointed out that there are eight dicot PM-specific CSEP genes that have a Ka/Ks ratio > 1, of which 6 are shared by *Gc*C1 and *Gc*M3, two most recently (1.3 MYA) diverged biotypes, suggesting that dicot PM-specific CSEP genes are also under strong positive selection and may contribute to host specialization relatively recently after speciation.

Notably, there is no significant difference between dicot and monocot PM fungi in terms of the number of genes encoding secreted proteins (SPs) that are not considered to be typical effectors [i.e. those classified as CAZymes and have homologs outside PM fungi] (Additional file 3: Table S22)]. Many of such SP genes encode digestive enzymes that may facilitate penetration of the host cell wall by PM pathogens, which is presumably important for both monocot and dicot PM fungi (Additional file 3: Table S22).

It is also worth noting that in this study we have followed the definition of CSEPs as secreted proteins that do not contain a transmembrane domain(s) and do not have homologs outside PM fungi (Spanu et al. [11]). However, this classification by nature is an underestimation for the real size of the effectoromes, and could also risk “losing” existing CESP genes when “homologs” are later found in other fungal species whose genomes have yet to be sequenced. For example, two PM CSEPs (OEC101 and OEC123) recently characterized from *G. orontii* [32] would not fit the “CSEP” category anymore, because now they could detect homologs in other fungal pathogens, such as *Oidiodendron maius*, and *Sclerotinia borealis*. Removing “no homology outside PM fungi” as a criterion for PM CSEPs, we could add 256–377 new CSEPs to the effectoromes of the eight PM genomes (Additional file 3: Table S22).

### Deciphering genes contributing to host adaptation

It is conceivable that SPs (of which CSEPs in particular) conserved in all PMs are more likely involved in basic invasive processes such as cell wall penetration, whereas LS or BS CSEPs are more likely to be involved in host-adaptation / specialization. Reliable computational prediction of effector genes is an important first step to investigate host-adaptation mechanisms of pathogenic fungi. The most important criterion for predicting an effector is that the candidate protein contains an N-terminal secretion signal. In this study, we used SignalP 3.0 [69] which is believed to be one of the most sensitive tools [70] to predict N-terminal secretion signals from genes of all eight PM genome to ensure comparability. Notably, while it was reported that ~ 80% (60–63% by our analysis) of the CSEPs encoded by the *Bgh* genome contain an Y/F/WxC (YxC for short) motif in the first 30 amino acids after the N-terminal signal peptide [11, 71], only 15 to 21 (i.e. 9–18%) CSEPs of the dicot PM biotypes possess an N-terminal YxC motif (Additional file 3: Table S23). Thus, it appears that YxC motif-containing CSEP genes must have been amplified in *Bgh* and *Bgt*, but this does not seem to have occurred in dicot PM fungi, highlighting a difference between the two PM lineages.

Conforming to the notion that PM effectors are evolving fast and thus few are shared between monocot PM and dicot PM [11], only eight CSEPs were found to be present in all eight PM genomes in our study (Fig. 3; Additional file 2: Table S6). It is conceivable that these eight core CSEPs and three likely core CSEPs may have an ancient origin and they probably play essential conserved roles in PM pathogenicity. This is consistent with a much lower Ka/Ks ratio for these eight CSEPs (0.03–0.43) compared to the average Ka/Ks ratio (0.72) for 249 LS CSEP gene clusters shared by dicot PM fungi or monocot PM fungi (Additional file 3: Table S24; Fig. 4c). In addition to eight core CSEP genes and three likely core CSEP genes that are present in seven of the eight PM genomes, there are probably additional “antique” CSEP genes falling into 24 gene clusters that may have evolved before the separation of monocot PM from dicot PM, because they are found in both monocot and dicot PM fungi albeit not in all of the eight PM genomes (Fig. 3). Loss of these genes in some but not all PM genomes suggests that their roles in pathogenicity are dispensable; however, it remains possible that failure in detecting some of these CSEP genes in all eight genomes could be due to insufficient sequence annotation, or insufficient sequence coverage and/or assembly.

Employing RNA-seq analysis to identify CSEPs that show preferential *in planta* expression could prioritize key CSEPs for functional characterization. In this study, we found that seven of the 81 *Gc*C1-unique CSEP genes are strongly up-regulated (3–263 fold) in haustoria formed in Arabidopsis (Additional file 3: Table S25), implying their potential involvement in *Gc*C1’s adaptation to Arabidopsis. Similarly, three *in planta* up-regulated CSEP genes shared by *Gc*M3 and *Gc*C1 (Additional file 3: Table S26) [two of which were also found in *G. orontii* [32]] but not by *Gc*M1 and *E. necator* may in part explain *Gc*M3’s ability to sporulate weakly on Arabidopsis leaves. In addition, four CSEP genes that showed *in planta* up-regulation in both *Gc*C1 and *Gc*M3 (note that they all have orthologs in *Gc*M1, and two of them also have homologs in *G. orontii* [32]) (Additional file 3: Table S27) may be good candidate effector genes responsible for overcoming penetration resistance in Arabidopsis, since all these PM biotypes can breach the leaf cell wall of Arabidopsis (Additional file 1: Figures S2-S4; Wen et al. [33]). Likewise, 18 *On*M2-specific CSEPs (Additional file 3: Table S28) which are up-regulated in haustoria may play crucial roles in *On*M2’s pathogenicity and could be prioritized for future functional studies through host-induced gene silencing [72, 73] and ectopic expression in host plants to see if they can support infection of an otherwise nonhost PM fungus such as *Gc*M1.

## Conclusions

Through a comprehensive genome analysis of four dicot PM fungi in comparison with those of two monocot PM *formae speciales*, we have revealed several new interesting features of PM genomes that may underscore their common mode of biotrophic parasitism as well as their contrasting host-adaptation specificities. First, despite a similar gene content, the genomes of PM fungi vary in genomes size, ranging from 120 to 222 Mb. This phenomenon is indicative of highly differential transposon activities in individual PM genomes, which also offers an explanation for common (hence ancient) as well as distinct (hence recent and even ongoing) gene losses in individual PM genomes. Second, while about 75% the total identified genes (including 3819 singleton gene clusters) are shared by all eight PM genomes, representing the core PM genome content responsible for basic functions of PM fungi, the remaining 25% are lineage specific or biotype specific, representing the variable PM genome content likely responsible for distinct host-adaptation. Third, compared to the effectoromes of monocot PM fungi (353–661 genes), dicot PM fungi have a much smaller effectorome (116–175 genes) with limited gene amplification. This sharp difference in effectorome size supports a hypothesis that while there has been an advanced arms race between monocots and their highly specialized PM fungi, the arms race between polyphagous dicot PM and their host plants has largely remained primitive.

## Methods

### Identification and maintenance of powdery mildew biotypes

We identified and collected three powdery mildew (PM) biotypes *Golovinomyces cichoracearum* (*Gc*) UMSG1, *Oidium neolycopersici* (*On*) UMSG2, and *Gc* UMSG3 respectively from plants of sow thistle (*Sonchus oleraceus*), tomato (*Solanum lycopersicum,* Moneymaker), and tobacco (*Nicotiana tobacum*) grown on the Shady Grove campus, University of Maryland. The other biotype *Gc* UCSC1 was obtained from the S. Somerville lab, University of California, Berkeley and maintained on susceptible Arabidopsis accession Col-0. All the three new PM biotypes were purified by inoculating clean host plants using spores from a single colony for more than three generations, then they were respectively maintained on sow thistle (for *Gc*M1), tomato (for *On*M2), or tobacco (for *Gc*M3) plants in separate plant growth chambers (8 h light at ~ 125 μmol^.^m^-2.^S^− 1^, 16 h dark). The identification of sow thistle and *Gc* UMSG1 was done by S. Xiao [33].

### Powdery mildew inoculation, and genomic DNA extraction

Seeds of Arabidopsis, tobacco, tomato, and sow thistle were sown on Sungro Horticulture Propagation Mix (Lot Code: INK 16071, Product of Canada) and were cold treated at 4 °C for 2~ 3 days, then germinated in a plant growth chamber under 22 °C, 75% relative humidity, short-day conditions (8 h light at ~ 125 μmol^.^m^-2.^S^− 1^, 16 h dark). Seedlings were grown under the same conditions for 6~ 8 weeks before powdery mildew inoculation. The four powdery mildew biotypes *Gc* UCSC1, *Gc* UMSG1, *On* UMSG2, and *Gc* UMSG3 were respectively inoculated on plants of Arabidopsis (Col-0), sow thistle, tomato, or tobacco and maintained in separate plant-growth chambers under similar growth conditions. At ~ 12 day-post-inoculation (dpi), infected leaves were collected and incubated at 37 °C for ~ 15 min. Powdery mildew spores were then harvested by gently brushing the spores off the leaves and filtering through a 50 μm mesh, and stored at − 80 °C. For extraction of total genomic DNA from powdery mildew spores, ~ 50 mg spores were mixed with 0.5 ml sterile nuclease-free sands (0.1 mm in diameter) and ground with a tissueLyser (Qiagen, Doncaster, Victoria) before DNA extraction and purification following the manufacturer’s instruction using the ZR fungal/Bacterial DNA Miniprep™ (Cat. No.: D6005, ZYMO Research).

### Genome sequencing and assembly

High quality DNA from fungal spores was used for DNA library preparation for sequencing using Illumina TruSeq™ DNA Sample Preparation Kit v2. Fragments with insert size around 500 bp were selected for sequencing on Illumina HiSeq1500 at the UM-IBBR Sequencing Core at the University of Maryland College Park. Sequenced reads were quality trimmed using Trimmomatic and adapters were removed with Cutadapt. High quality reads were assembled using CLC Workbench v6.1 (http://www.clcbio.com/products/clc-genomics-workbench/). All assemblies were generated using a word size of 24 and a bubble size of 50. Genome size of the PM fungi was estimated based on k-mer count distribution from JELLYFISH version 1.1.11 [74]. The completeness of DNA sequence scaffolds was evaluated using Eukaryotic Genes Mapping Approach (CEGMA) analysis and BUSCO [35, 75].

### Annotation of repetitive sequences

Repeat sequences, including transposon elements (TEs), simple repeat and low complexity sequences were classified and annotated using RepeatModeler followed by RepeatMasker. First, RepeatModeler (version 1.0.8) was used to perform ab initio repeat prediction and then the libraries of consensuses representing TE families characterized from RepeatModeler were classified on CENSOR website (http://www.girinst.org/censor/) and used to screen the TE copies from each scaffold using RepeatMasker (version 3.3.0) (http://www.repeatmasker.org/).

### RNA extraction and transcriptome sequencing

To obtain gene expression data from mycelia for evidence-based gene prediction and annotation, and for comparative transcriptome analysis between haustoria and mycelia, we collected mycelia from all the four dicot PM fungi as described below. At 6 dpi when an extensive mycelial network is established while sporulation is still at a low level, the infected leaves were subjected to spore-removing in a flow hood using pressured air to blow the leaves gently and thoroughly. This treatment can remove most mature spores as shown under a microscope (Additional file 1: Figure S11A). The mycelial network containing conidiophores were collected by gently brushing them off the leaf surface using TRIzol reagent (Cat. No. 15596026, Thermo Fisher Scientific). The mycelia in TRIzol were pooled in a 1.5 ml tube for RNA extraction. For preparing leaves containing haustoria, infected leaves were cut at 6 dpi and gently washed with ddH_2_O to remove all mycelia and spores (Additional file 1: Figure S11B), frozen in liquid N_2_ and stored at − 80 °C for extraction of total plant and fungal RNA. RNA extraction and purification from mycelia or haustoria-containing host cells was conducted using TRIzol reagent (Cat. No. 15596026, Thermo Fisher Scientific) after grinding harvested mycelia or leaf tissue with a pestle in a 1.5 ml tube.

For RNA-seq, we prepared three replicates of total RNA sample from mycelia for each of the four dicot PM biotypes, and three replicates of total RNA samples from haustoria-containing leaf tissues for *Gc*M3, *On*M2 and *Gc*C1. The cDNA libraries were prepared using Illumina TruSeq™ RNA Sample Preparation Kit v2 and transcriptome sequencing was performed on Illumina Hiseq 1500 at the UM-IBBR Sequencing Core at the University of Maryland. Sequence reads were quality filtered using following criteria (the perl scripts for quality filter was uploaded to github (https://github.com/wuying1984/genomics_related_scripts/blob/master/perl%20scripts%20for%20quality%20filter)):

1. average quality score >20
2. no N in the first 20 bp
3. >50% of the nucleotides with quality <5
4. >20% of the nucleotides with quality <13
5. >10% of the nucleotides with quality <10

### Gene prediction and functional annotation

Gene structural prediction was performed using MAKER2 [42] in order to combine results from: 1) three ab initio prediction using SNAP [76], AUGUSTUS [77], and GeneMark [78]; 2) homology based comparison against Uniprot protein database and available proteins of *Bgh*D (https://genome.jgi.doe.gov/Blugr2/Blugr2.home.html), *Bgt* (https://www.ncbi.nlm.nih.gov/genome/845?genome_assembly_id=300412), *E. necator* (https://www.ncbi.nlm.nih.gov/genome/35332) and *G. orontii* [32]; and 3) transcriptome analysis [i.e. EST assembled from mycelia and haustoria RNA-seq reads for each biotypes using Trinity [79]. We performed complete de novo gene annotations for all the four dicot PM fungi. In the first run, the ab initio gene prediction software was trained using core ascomycota orthologs identified by BUSCO [75] and the est2genome and protein2genome were set to 1. Next, the resulting gene models from the first MAKER run were used to train SNAP for the second run. And then the annotation pipeline was run a third time for generating the final gene models for each biotype. Since effector gene structure is quite different from canonical conserved fungal genes, we therefore trained SNAP and AUGUSTUS using all the genes predicted to encode secreted proteins (SPs) from previous three iterative runs. Then we added these SP gene models to MAKER and performed two more MAKER runs in order to identify all potential genes encoding CSEPs. All the predicted genes were manually curated using Web Apollo [80]. The complete protocol for the five round of MAKER prediction is uploaded to github (https://github.com/wuying1984/MAKER2_PM_genome_annotation). The final proteome from each biotype were assessed against the Ascomycota database (ascomycota_odb9) for completeness. Overall, the gene annotation has a very high quality (~ 97% BUSCO coverage for each of the four dicot PM protein sets; Additional file 3: Table S29). A predicted gene lacking a start and/or a stop codon is marked as “truncated” in each genome. Functional annotation of proteins was carried out using InterProScan 5 [46].

### Identification of secreted proteins and candidate effectors

Secreted proteins (SPs) were predicted using a combination of classical predictors: SignalP 3.0 for peptide signals [69] and TMHMM 2.0c for transmembrane domains [81]. Those secreted proteins that lack a transmembrane domain in mature proteins and have no hit outside powdery mildew (BLASTP e-value < 10^− 10^) are defined as candidate secreted effector proteins (CSEPs). Since CSEPs are not easy to predict because they tend to be short and have few homologs, we also performed ORF (open reading frame) prediction on the scaffolds using getorf in the EMBOSS package [82]. All predicted ORFs with an N-terminal secretion signal and gene expression evidence were identified as genes encoding secreted proteins.

### Gene cluster analysis

Gene clusters were characterized using OrthoFinder [83] with default parameters except for choosing identity cutoff of 0.3. All genes were clustered into the following categories: 1) Core cluster: gene clusters containing members from all eight PM genomes; 2) Likely core cluster: gene clusters containing members from 7 of the 8 genomes; 3) Dicot PM-specific cluster: gene clusters containing members only from the dicot PM biotypes; 4) Monocot PM-specific cluster: gene clusters containing members only from the monocot PM biotypes; 5) Other: gene clusters containing members from 2 to 6 PM genomes including both dicot- and monocot-PM biotypes; 5) BS genes: genes that cannot be clustered with genes from other genomes. The script for gene conservation definition has been uploaded to github (https://github.com/wuying1984/genomics_related_scripts/blob/master/1get_group_conserve.pl). All other the scripts developed in this work but not deposited in github will be provided upon request.

Protein sequences in each cluster (only *Bgh*D protein sequences were used to represent *Bgh* biotype) were aligned, which further guided the alignments of the corresponding coding sequences. The Ka/Ks ratio of each coding sequence alignment was processed using codeml program of PAML package [84].

### Gene expression analysis

The expression level of each gene was determined in mycelia for all 4 PM biotypes and haustoria for *Gc* UCSC1, *On* UMSG2 and *Gc* UMSG3 using the RNA-seq data. Tophat 2.0.13 [85] was used to map RNA-seq reads to the genomic scaffolds. And then cuffdiff [86] was used to compare the expression levels between haustoria and mycelia. Genes with a FPKM (Fragments Per Kilobase of transcript per Million mapped reads) > 1.5 was defined as expressed. All genes with a fold change > 2 and q-value (adjusted *p*-value) < 0.05 were defined as differentially expressed.

### Phylogenetic tree construction and divergence time estimation

To estimate divergence time among the seven PM biotypes (including two isolates of *Bgh*), *Neurospora crassa* were used as an outgroup. Protein sequences from *N. crassa* (GCA_000182925.2) were downloaded from Genbank and used for identifying the best conserved orthologs for the 3819 gene clusters (BLASTP with e-value < 10^− 10^ & sequence identity > 50%). The final data set was composed of 1716 orthologous genes from *N. crassa* and the eight PM genomes. The protein sequences in each clusters were aligned using the T_Coffee software [87]. Then the alignment of the corresponding coding sequences (CDS) was obtained using perl script. Gblocks was used to filter putative nonreliable positions [88]. All clusters were concatenated into one fasta file for phylogenetic analysis. The phylogenetic tree was constructed using MEGA 7.0 with Neighbor-joining method and bootstrap 1000. Then the phylogenetic tree was calibrated by setting the divergence time between *Bgh* and *Bgt* to 5.2–7.4 million years ago [12, 19].

## Additional files

Additional file 1: **Figure S1.** A phylogenetic tree with divergence time of the eight powdery mildew (PM) genomes. **Figure S2.** *Oidium neolycopersici* UMSG2 (*On*M2) has a broad host range. **Figure S3.** *Golovinomyces cichoracearum* UCSC1 (*Gc*C1) is infectious on Arabidopsis (A, B, D), cucurbits (e.g. squash; C). **Figure S4.** *Golovinomyces cichoracearum* UMSG3 (*Gc*M3) sporulates heavily on tobacco and *Nicotiana benthamiana* (A,B), rarely on wild-type Arabidopsis (C,E) but profusely on Arabidopsis *pad4/sid2* mutant plants (D,F). **Figure S5.** Percentage of powdery mildew (PM) genes with unknown function or without homologs outside PM in the NCBI NR database (E value < 10^− 10^) from different cluster categories. **Figure S6.** A Comparative Gene Ontology (GO) term enrichment analysis for lineage-specific genes from dicot PM fungi (Dicot PM LS gene) (A) and monocot PM fungi (Monocot PM LS gene) (B). **Figure S7.** Differential expansion of genes encoding secreted proteins (SP) or candidate secreted effector proteins (CSEP) of the eight powdery mildew biotypes. **Figure S8.** Comparison of gene expression between different replicates of haustorial RNA samples (H_rep1 to H_rep3) and between these H samples and the spores/mycelial RNA samples (M) of the tomato PM biotype *Oidium neolycopersici* UMSG2 (*On*M2). **Figure S9.** Comparison of gene expression between different replicates of haustorial RNA samples (H_rep1 to H_rep3) and between these H samples and the spores/mycelial RNA samples (M) of the tobacco PM biotype *Golovinomyces cichoracearum* UMSG3 (*Gc*M3). **Figure S10.** Frequency of predicted standard genes with both start and stop codons (denoted as “Complete”) and partial genes missing the start and/or the stop codon (denoted as “Partial”) that have been mapped to assembled scaffolds relative to the scaffold ends. **Figure S11.** Preparation of mycelial and haustorial samples for RNA-seq analysis. (PDF 9935 kb)

Additional file 2: **Table S1.** Transposable elements (TEs) in the assembled genome of each powdery mildew biotype. **Table S2.** Distribution of genes controlling proliferation of repetitive sequences in the PM genomes. **Table S3.** Percentage of PM genes with homologs in other fungi. **Table S4.** Ascomycete core genes missing in each PM biotype and three other obligate biotrophic fungi. **Table S5.** Gene clusters grouped by all genes from the eight powdery mildew genomes. **Table S6.** Grouping of protein-encoding genes of eight sequenced PM genomes. **Table S7.** Number of gene clusters with no homologs outside PM genomes. **Table S8.** Top 100 biological processes defined by the 3819 core genes from eight PM genomes via Gene Ontology analysis. **Table S9.** Genes involved in secondary metabolism from eight dicot PM genomes. **Table S10.** Genes encoding carbohydrate-active enzymes (CAZy) in eight PM genomes. **Table S11.** Coding nucleotide and the corresponding amino acid sequences of genes predicted to encode secreted proteins or CSEPs from four dicot PM biotypes. **Table S12.** RNase-like genes in the eight PM genomes. (XLSX 1897 kb)

Additional file 3: **Table S13.** Mapping of RNA-seq reads to two PM genomes. **Table S14.** Percentages of PM genes expressed in mycelia and/or haustoria. **Table S15.** Functional enrichment of the differentially expressed genes in *Oidium neolycopersici* UMSG2. **Table S16.** Functional enrichment of the differentially expressed genes in *Golovinomyces cichoracearum* UMSG3. **Table S17.** Orthologous genes up-regulated between any two of the three PM biotypes examined. **Table S18.** Orthologus genes Up-regulated in haustoria in both *On*M2 and *Gc*M3. **Table S19.** Orthologus genes Up-regulated in haustoria in both *Gc*C1 and *Gc*M3. **Table S20.** Total number and percentage of predicted partial genes that are expressed. **Table S21.** A comparison of powdery mildew interaction with dicot and monocot hosts. **Table S22.** Composition of genes defining the secretome in eight PM genomes. **Table S23.** Predicted powdery mildew genes encoding proteins with a YxC motifa in eight PM genomes. **Table S24.** Ka/Ks ratios of CSEP gene clusters from seven PM biotypes. **Table S25.** High-level expression of six *G. cichoracearum* UCSC1-specific CSEP genes in haustoria. **Table S26.** CSEP genes of *Gc* UCSC1 and *Gc* UMSG3 that show induced expression in haustoria. **Table S27.** Shared CSEP genes among *Gc* UCSC1, *Gc* UMSG3 and *Gc* UMSG1 that show induced expression in haustoria of *Gc* UCSC1 and *Gc* UMSG3. **Table S28.** *O. neolycopersici* UMSG2-specific CSEP genes that are induced in haustoria. **Table S29.** BUSCO completeness assessment of the protein set for each PM biotype. (XLSX 234 kb)

## Abbreviations

ACGs: ascomycete core genes
BS: biotype-specific
CAZy: carbohydrate-active enzymes
CDS: coding sequences
CEGs: core eukaryotic genes
CSEP: candidate secreted effector protein
ETI: effector-triggered immunity
Ka: nonsynonymous substitution rate
Ks: synonymous substitution rate
LRR: leucine-rich repeat
LS: lineage-specific
NB: nucleotide binding
NGS: next-generation sequencing
PAMP: pathogen-associated molecular patterns
PM: powdery mildew
PTI: PAMP-triggered immunity
SP: secreted proteins
