# Supplementary material for: Comparative genome analyses reveal sequence features reflecting distinct modes of host-adaptation between dicot and monocot powdery mildew
Source: BMC Genomics. 2018 Sep 25;19:705. doi: 10.1186/s12864-018-5069-z (PMC6156980; doi:10.1186/s12864-018-5069-z)
Supplement: Supplementary file 1 — Figure S1. A phylogenetic tree with divergence time of the eight powdery mildew (PM) genomes. Figure S2. Oidium neolycopersici UMSG2 (OnM2) has a broad host range. Figure S3. Golovinomyces cichoracearum UCSC1 (GcC1) is infectious on Arabidopsis (A, B, D), cucurbits (e.g. squash; C). Figure S4. Golovinomyces cichoracearum UMSG3 (GcM3) sporulates heavily on tobacco and Nicotiana benthamiana (A,B), rarely on wild-type Arabidopsis (C,E) but profusely on Arabidopsis pad4/sid2 mutant plants (D,F). Figure S5. Percentage of powdery mildew (PM) genes with unknown function or without homologs outside PM in the NCBI NR database (E value < 10− 10) from different cluster categories. Figure S6. A Comparative Gene Ontology (GO) term enrichment analysis for lineage-specific genes from dicot PM fungi (Dicot PM LS gene) (A) and monocot PM fungi (Monocot PM LS gene) (B). Figure S7. Differential expansion of genes encoding secreted proteins (SP) or candidate secreted effector proteins (CSEP) of the eight powdery mildew biotypes. Figure S8. Comparison of gene expression between different replicates of haustorial RNA samples (H_rep1 to H_rep3) and between these H samples and the spores/mycelial RNA samples (M) of the tomato PM biotype Oidium neolycopersici UMSG2 (OnM2). Figure S9. Comparison of gene expression between different replicates of haustorial RNA samples (H_rep1 to H_rep3) and between these H samples and the spores/mycelial RNA samples (M) of the tobacco PM biotype Golovinomyces cichoracearum UMSG3 (GcM3). Figure S10. Frequency of predicted standard genes with both start and stop codons (denoted as “Complete”) and partial genes missing the start and/or the stop codon (denoted as “Partial”) that have been mapped to assembled scaffolds relative to the scaffold ends. Figure S11. Preparation of mycelial and haustorial samples for RNA-seq analysis. (PDF 9935 kb) [file 12864_2018_5069_MOESM1_ESM.pdf]

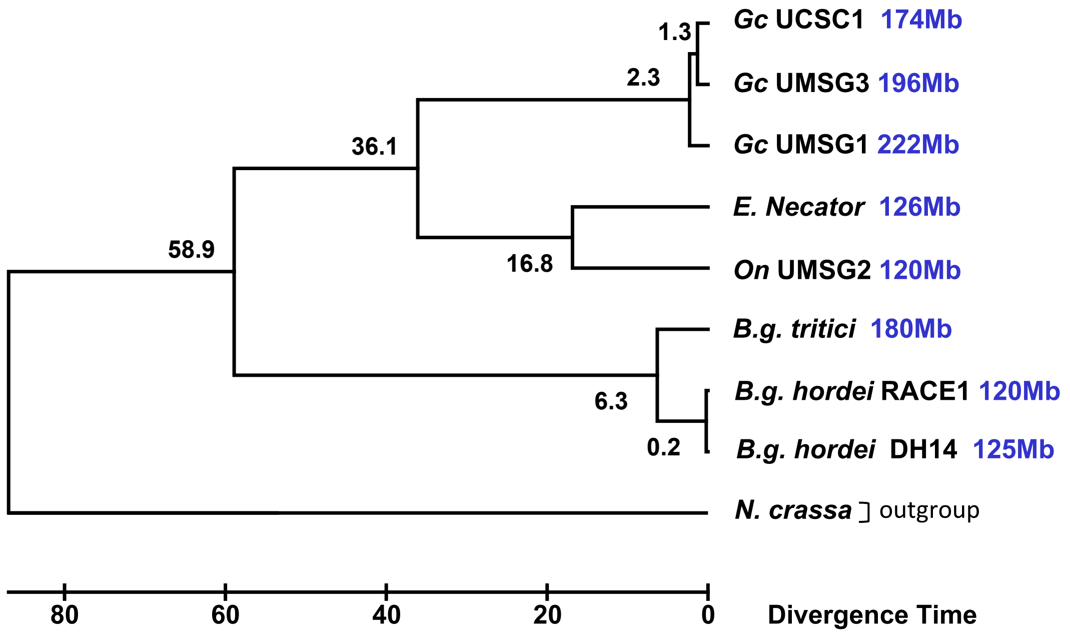

Supplemental Figure S1. A phylogenetic tree with divergence time of the eight powdery mildew (PM) genomes. 1716 genes from the 3819 core PM gene clusters of the eight PM genomes that have conserved orthologs in *Neurospora crassa* (identity >0.5 & e-value <10^{-10} at the protein level) were used for the phylogenetic analysis. The tree was constructed using the Maximum Likelihood method based on the Hasegawa-Kishino-Yano model [1] performed with MEGA7.0 [2]. Only nucleotides in the third codon position were used for this analysis. Positions with less than 95% site coverage were eliminated. These include positions with fewer than 5% alignment gaps, missing data, and ambiguous bases. The divergence time shown in the tree was calculated using the RelTime method [3] and was calibrated by setting the divergence time between *Bgh* and *Bgt* to 5.2 to 7.4 million years ago [4]. The tree is drawn to scale in divergence time with branch lengths measured in the relative number of substitutions per site.

1. Hasegawa M., Kishino H., and Yano T. (1985). Dating the human-ape split by a molecular clock of mitochondrial DNA. J Mol Evol.22:160-174.2.
2. Kumar S., Stecher G., and Tamura K. (2015). MEGA7: Molecular Evolutionary Genetics Analysis version 7.0 for bigger datasets. Mol Bio Evol. 33:1870-1874.
3. Tamura K., Battistuzzi FU, Billing-Ross P, Murillo O, Filipski A, and Kumar S. (2012). Estimating Divergence Times in Large Molecular Phylogenies. Proc Natl Acad Sci. 109:19333-19338.
4. Wicker T, Oberhaensli S, Parlange F, Buchmann JP, Shatalina M, Roffler S, Ben-David R., Dolezel J., Simkova H., Schulze-Lefert P. et al. (2013). The wheat powdery mildew genome shows the unique evolution of an obligate biotroph. *Nat Genet* **45**(9): 1092-1096.

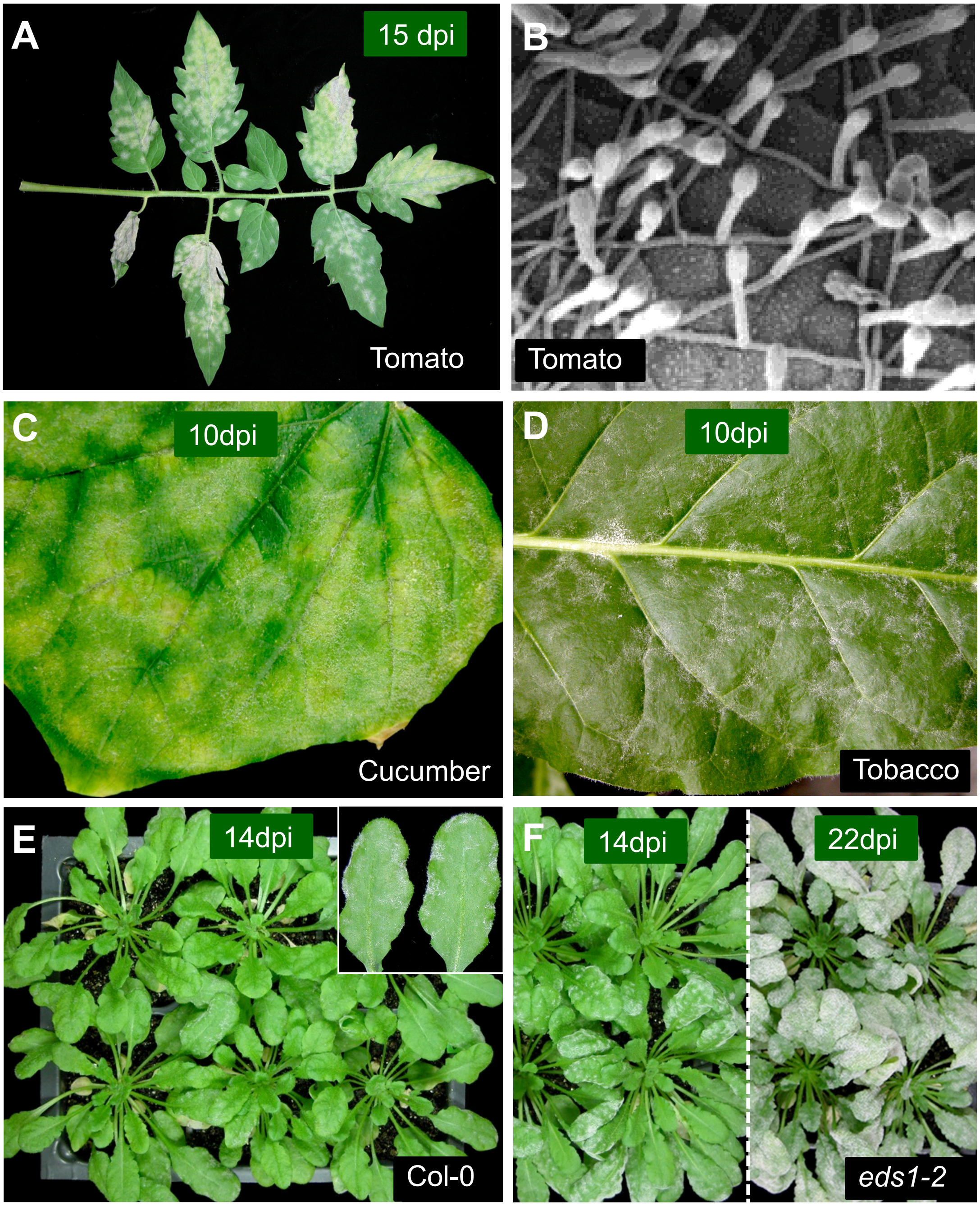

Supplemental Figure S2. *Oidium neolycopersici* UMSG2 (*On*M2) has a broad host range. It is infectious on tomato (**A**,**B**), cucumber (**C**), tobacco (**D**), and Arabidopsis (**E**,**F**). Note, *On*M2 only sporulates lightly on Col-0 WT (**E**), but profusely on *eds1-2* (**F**).

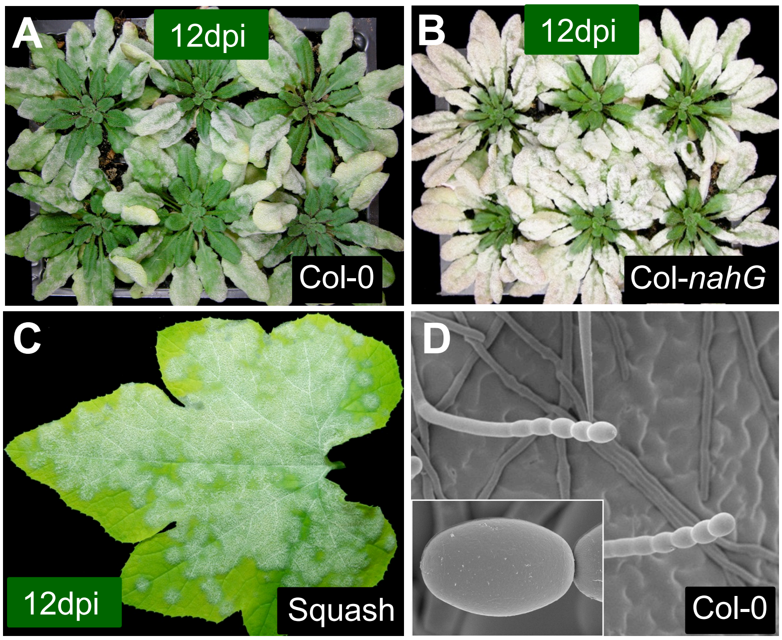

Supplemental Figure S3. *Golovinomyces cichoracearum* UCSC1 (*Gc*C1) is infectious on Arabidopsis (**A, B, D**), cucurbits (e.g. squash; **C**) and tobacco (Xiao et. al., 2003. MPMI 16, 289-294).

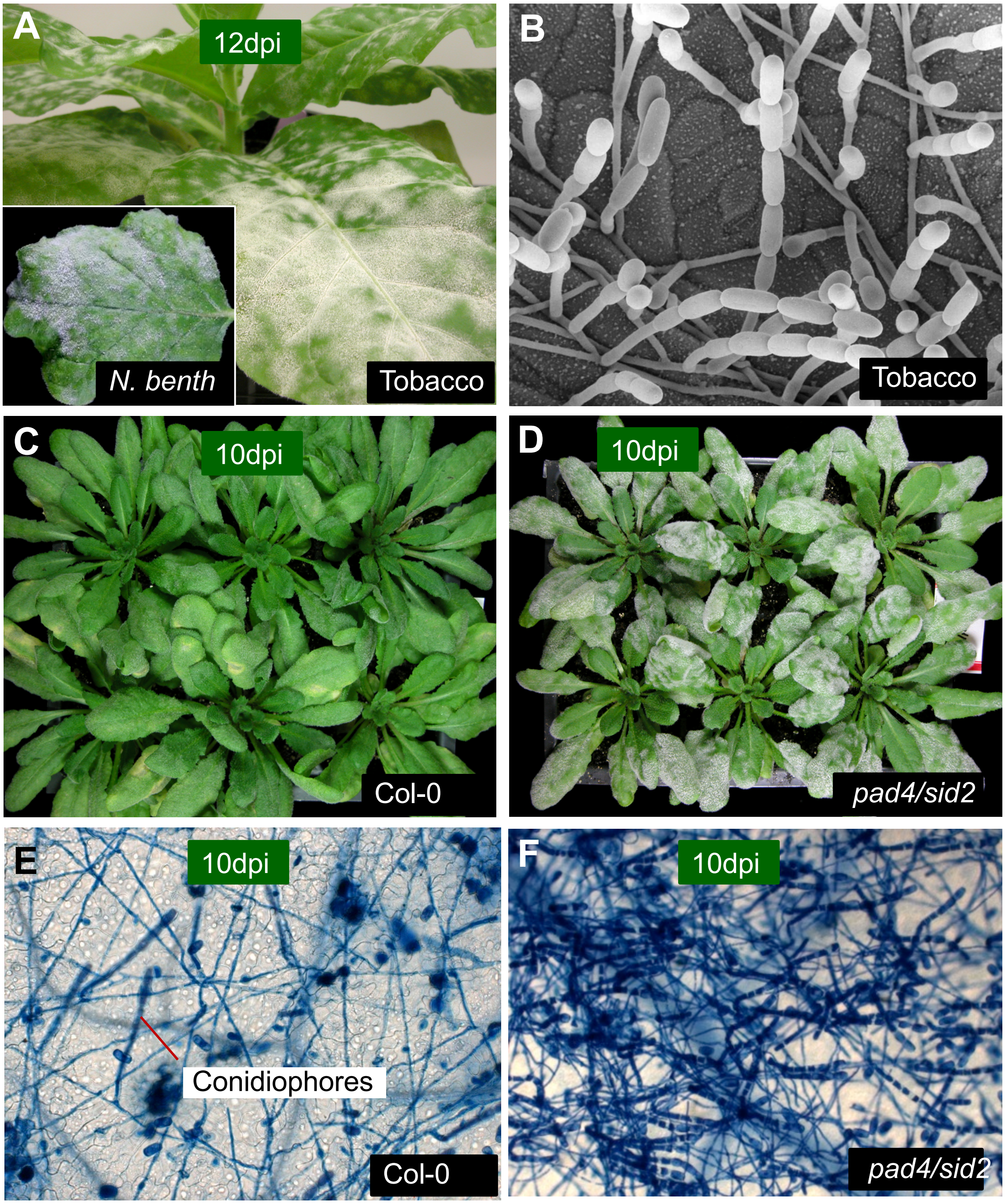

Supplemental Figure S4. *Golovinomyces cichoracearum* UMSG3 (*Gc*M3) sporulates heavily on tobacco and *Nicotiana benthamiana* (**A,B**), rarely on wild-type Arabidopsis (**C,E**) but profusely on Arabidopsis *pad4/sid2* mutant plants (**D,F**).

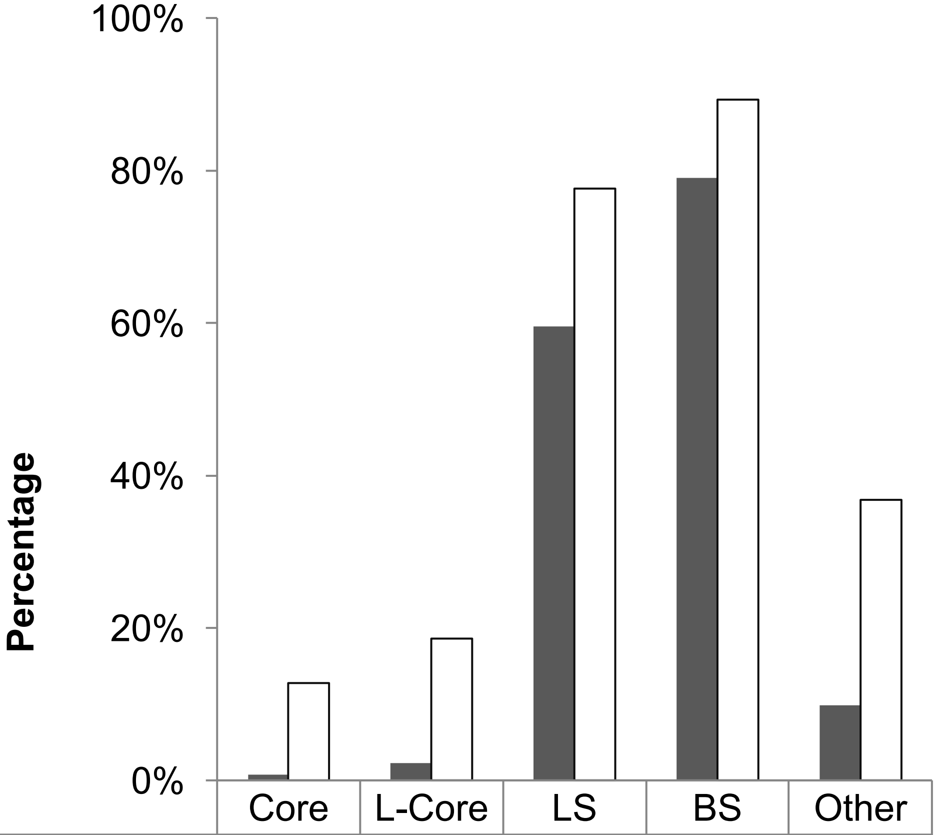

	Core	L-Core	LS	BS	Other
■ Without homolog outside PM	0.74%	2.26%	59.53%	79.03%	9.81%
□ with unknown function	12.76%	18.55%	77.59%	89.25%	36.84%

Supplemental Figure S5. Percentage of powdery mildew (PM) genes with unknown function or without homologs outside PM in the NCBI NR database (E value < 10^{-10}) from different cluster categories. Core, core gene clusters; L-core, Likely core gene clusters; LS, lineage-specific gene clusters; BS, Biotype-specific gene clusters; Other, the remaining gene clusters.

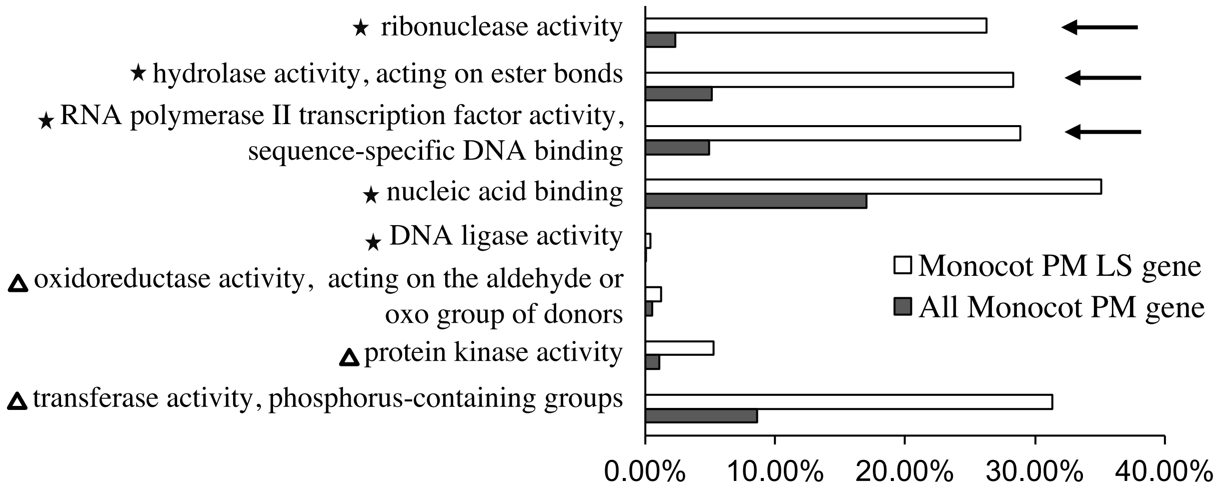

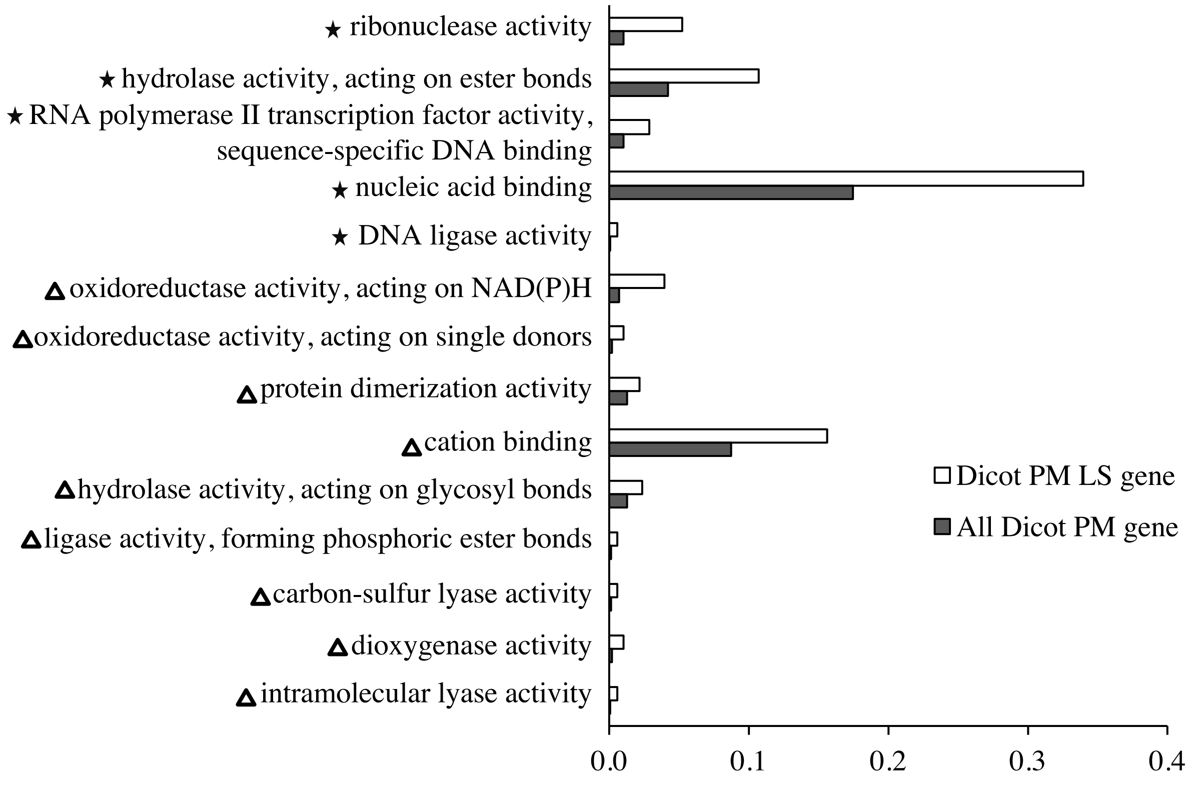

Supplemental Figure S6. A Comparative Gene Ontology (GO) term enrichment analysis for lineage specific genes from dicot PM fungi (Dicot PM LS gene) (A) and monocot PM fungi (Monocot PM LS gene) (B). Level three molecular function terms which have at least 1.5x enrichment in lineage specific genes compared to the total respective dicot or monocot PM genes were presented. Asterisks indicate significant enrichment in both Monocot PM SP and Dicot PM LS genes; Triangles indicate significant enrichment in either Monocot PM LS genes or Dicot PM LS genes. Arrows indicate more significant enrichment in the LS genes of dicot PM lineages compared to those of monocot PM lineages or vice versa.

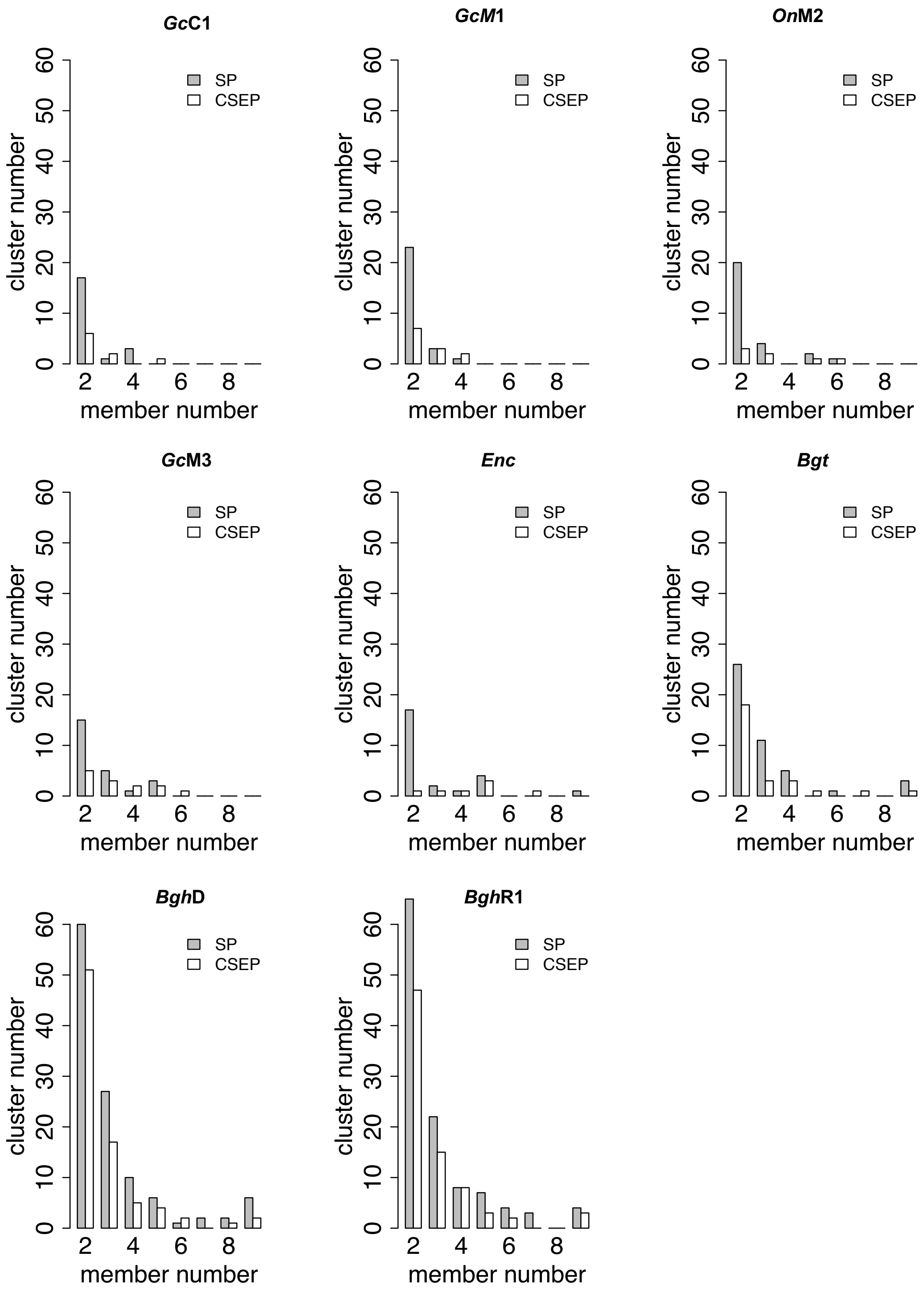

Supplemental Figure S7. Differential expansion of genes encoding secreted proteins (SP) or candidate secreted effector proteins (CSEP) of the eight powdery mildew biotypes. The x-axis denotes the size of a gene family (i.e. the number of family members), while the y-axis denotes the number of gene clusters with gene expansion. *Gc*C1, *Golovinomyces cichoracearum* UCSC1; *Gc*M1, *G. cichoracearum* UMSG1; *On*M2, *Oidium neolycopersici* UMSG2; *Gc*M3, *G. cichoracearum* UMSG3. *BghD*, *Blumeria graminis* f.sp. *hordei DH14*; *Bgh*R1, *B. graminis* f.sp. *hordei RACE1*; Bgt, *Blumeria graminis* f.sp. *tritici*.

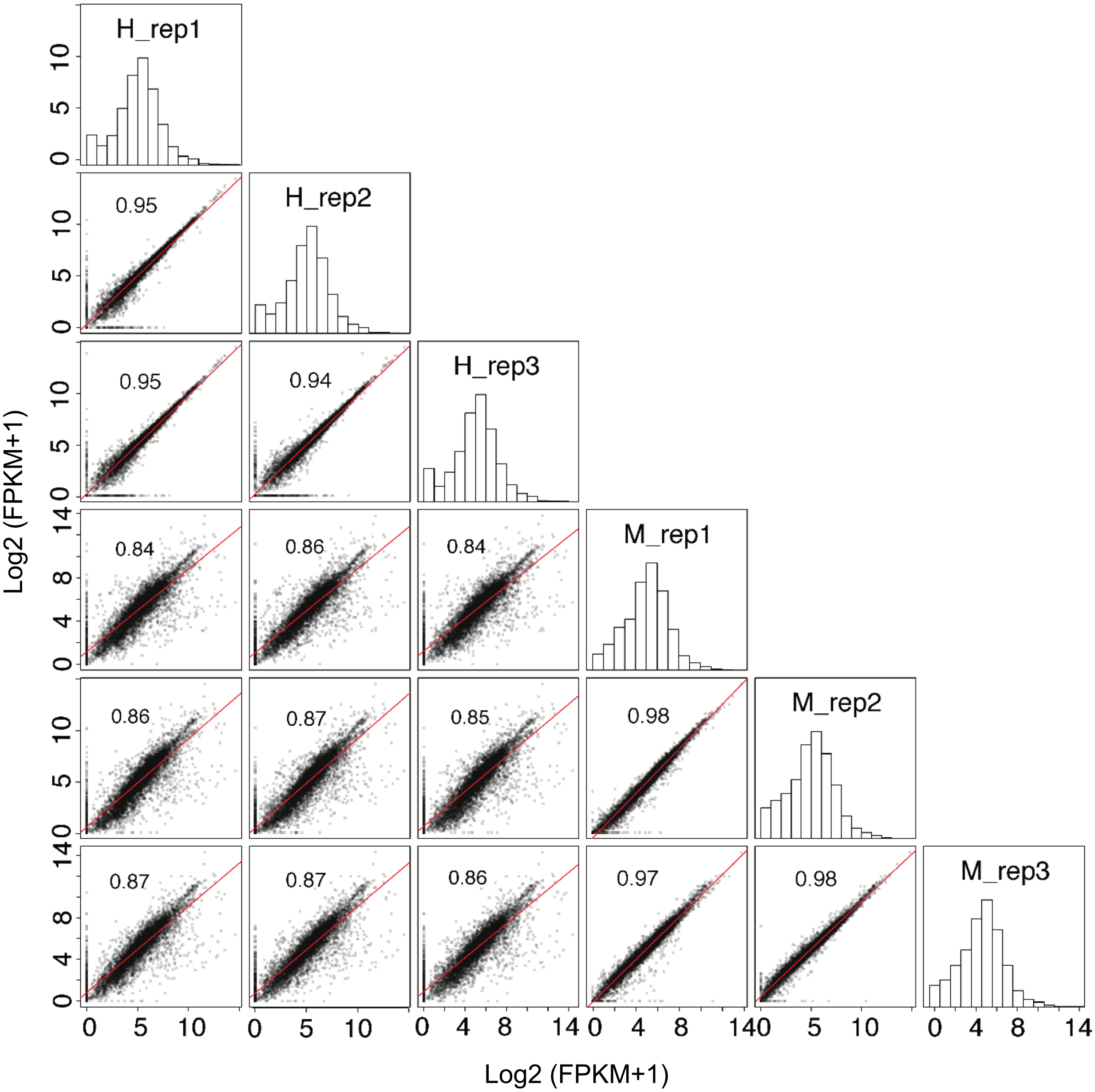

Supplemental Figure S8. Comparison of gene expression between different replicates of haustorial RNA samples (H_rep1 to H_rep3) and between these H samples and the spores/mycelial RNA samples (M) of the tomato PM biotype *Oidium neolycopersici* UMSG2 (*On*M2).

The histograms depict the gene expression profile of all genes in log2(FPKM+1) values of the M and the three H samples. The numbers denote the correlation efficiency of log2(FPKM+1) between indicated M and H samples, and between different H samples or M samples. The dot plot graphs represent the correlation between each indicated RNA samples. X-axis and Y-axis are the log2(FPKM+1) value in each sample.

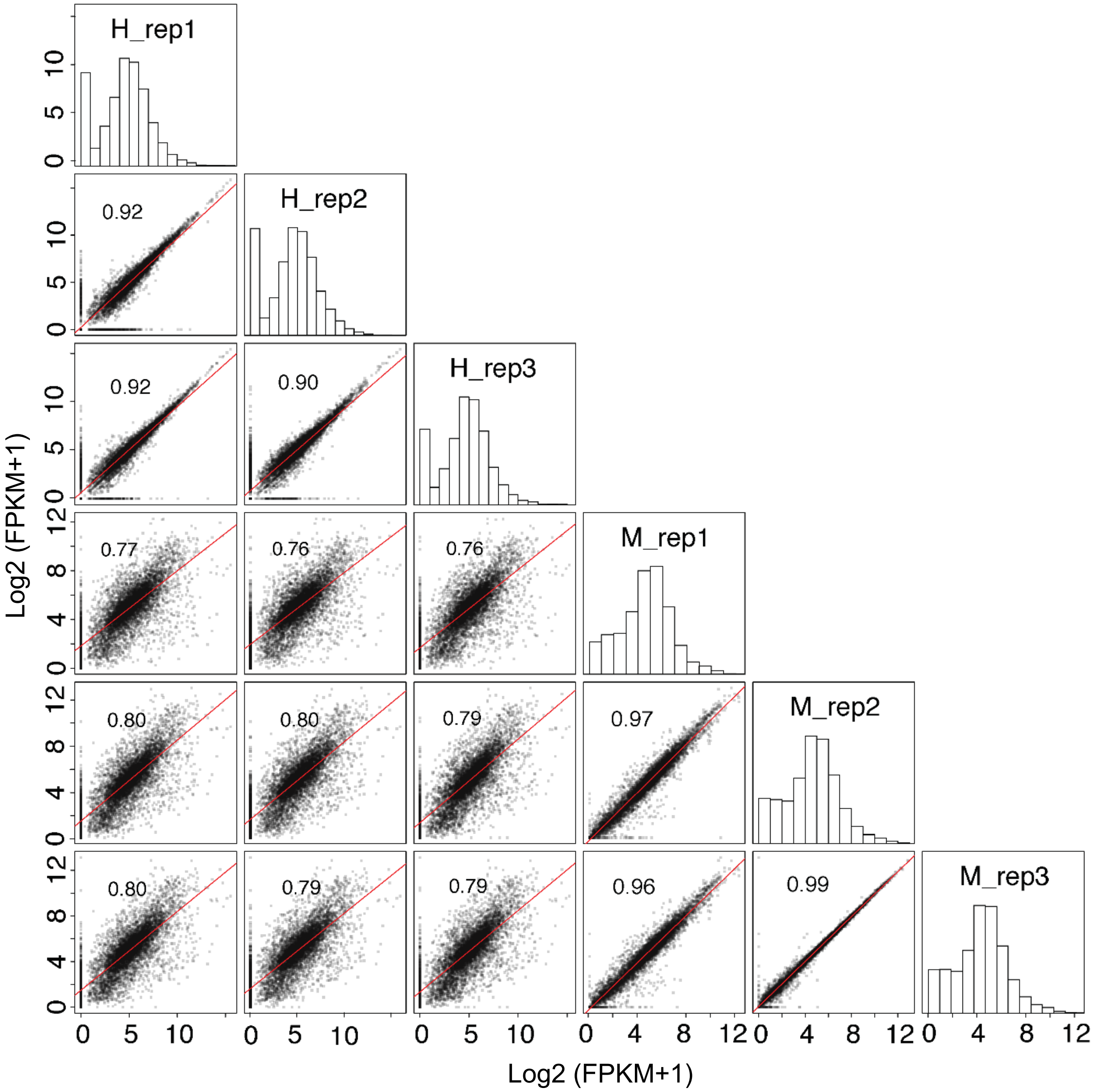

Supplemental Figure S9. Comparison of gene expression between different replicates of haustorial RNA samples (H_rep1 to H_rep3) and between these H samples and the spores/mycelial RNA samples (M) of the tobacco PM biotype *Golovinomyces cichoracearum* UMSG3 (*Gc*M3).

The histograms depict the log2 (FPKM+1) values in the M and the three H samples. The numbers denote the correlation efficiency of log2(FPKM+1) between the M and the indicated H samples, and between different H samples. The dot plot graphs represent the correlation between each indicated RNA samples. X-axis and Y-axis are the log2(FPKM+1) value in each sample.

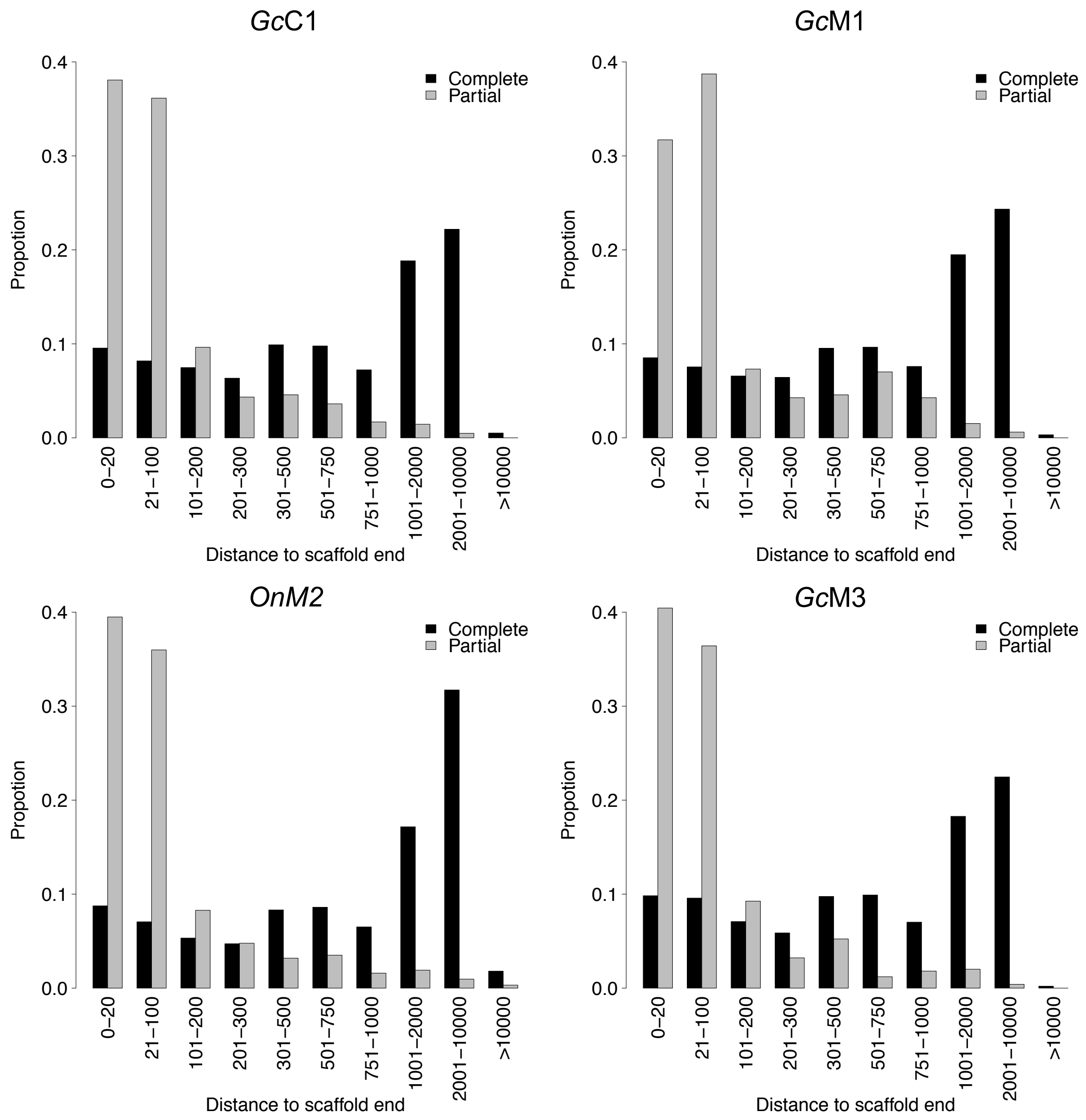

Supplementary Figure S10. Frequency of predicted standard genes with both start and stop codons (denoted as "Complete") and partial genes missing the start and/or the stop codon (denoted as "Partial") that have been mapped to assembled scaffolds relative to the scaffold ends.
*Gc*C1, *Golovinomyces cichoracearum* UCSC1; *Gc*M1, *G. cichoracearum* UMSG1; *On*M2, *Oidium neolycopersici* UMSG2; *Gc*M3, *G. cichoracearum* UMSG3.

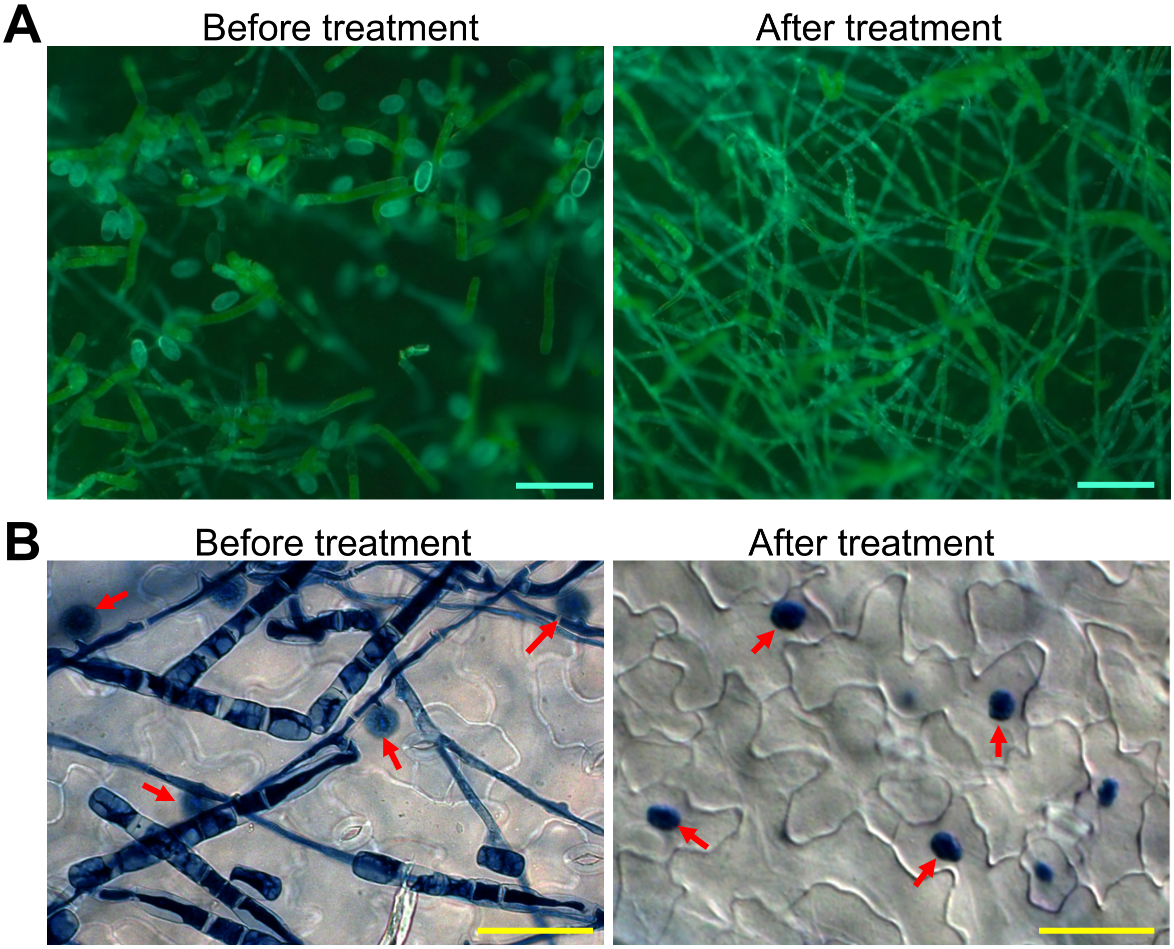

Supplementary Figure S11. Preparation of mycelial and haustorial samples for RNA-seq analysis. (A) Representative microscopic images showing fungal structures on the surface of an Arabidopsis leaf infected with *Gc* UCSC1 at 6 dpi before and after blowing away mature spores using pressure air in a flow hood. Fungal structures were stained with DiOC6. (B) Representative microscopic images showing fungal structures on the surface of an Arabidopsis leaf infected with *Gc* UCSC1 at 6 dpi before and after removing mycelia and spores by gentle brushing leaves with running water. Fungal structures were stained with trypan blue. Arrows indicate fungal haustoria. Bars = 100 µm in (A) and 50 µm in (B).
